# Skin-penetrating nematodes exhibit life-stage-specific interactions with host-associated and environmental bacteria

**DOI:** 10.1186/s12915-021-01153-7

**Published:** 2021-10-07

**Authors:** Ivan N. Chavez, Taylor M. Brown, Adrien Assié, Astra S. Bryant, Buck S. Samuel, Elissa A. Hallem

**Affiliations:** 1 Department of Microbiology, Immunology, and Molecular Genetics, University of California, Los Angeles, Los Angeles, CA 90095 USA; 2 Molecular Biology Institute, University of California, Los Angeles, Los Angeles, CA 90095 USA; 3 Alkek Center for Metagenomics and Microbiome Research, Baylor College of Medicine, Houston, TX 77030 USA; 4 Department of Molecular Virology and Microbiology, Baylor College of Medicine, Houston, TX 77030 USA

**Keywords:** *Strongyloides*, Parasitic nematode, Chemosensation, Sensory behavior, Bacteria

## Abstract

**Background:**

Skin-penetrating nematodes of the genus *Strongyloides* infect over 600 million people, posing a major global health burden. Their life cycle includes both a parasitic and free-living generation. During the parasitic generation, infective third-stage larvae (iL3s) actively engage in host seeking. During the free-living generation, the nematodes develop and reproduce on host feces. At different points during their life cycle, *Strongyloides* species encounter a wide variety of host-associated and environmental bacteria. However, the microbiome associated with *Strongyloides* species, and the behavioral and physiological interactions between *Strongyloides* species and bacteria, remain unclear.

**Results:**

We first investigated the microbiome of the human parasite *Strongyloides stercoralis* using 16S-based amplicon sequencing. We found that *S. stercoralis* free-living adults have an associated microbiome consisting of specific fecal bacteria. We then investigated the behavioral responses of *S. stercoralis* and the closely related rat parasite *Strongyloides ratti* to an ecologically diverse panel of bacteria. We found that *S. stercoralis* and *S. ratti* showed similar responses to bacteria. The responses of both nematodes to bacteria varied dramatically across life stages: free-living adults were strongly attracted to most of the bacteria tested, while iL3s were attracted specifically to a narrow range of environmental bacteria. The behavioral responses to bacteria were dynamic, consisting of distinct short- and long-term behaviors. Finally, a comparison of the growth and reproduction of *S. stercoralis* free-living adults on different bacteria revealed that the bacterium *Proteus mirabilis* inhibits *S. stercoralis* egg hatching, and thereby greatly decreases parasite viability.

**Conclusions:**

Skin-penetrating nematodes encounter bacteria from various ecological niches throughout their life cycle. Our results demonstrate that bacteria function as key chemosensory cues for directing parasite movement in a life-stage-specific manner. Some bacterial genera may form essential associations with the nematodes, while others are detrimental and serve as a potential source of novel nematicides.

**Supplementary Information:**

The online version contains supplementary material available at 10.1186/s12915-021-01153-7.

## Background

**Fig. 1:**
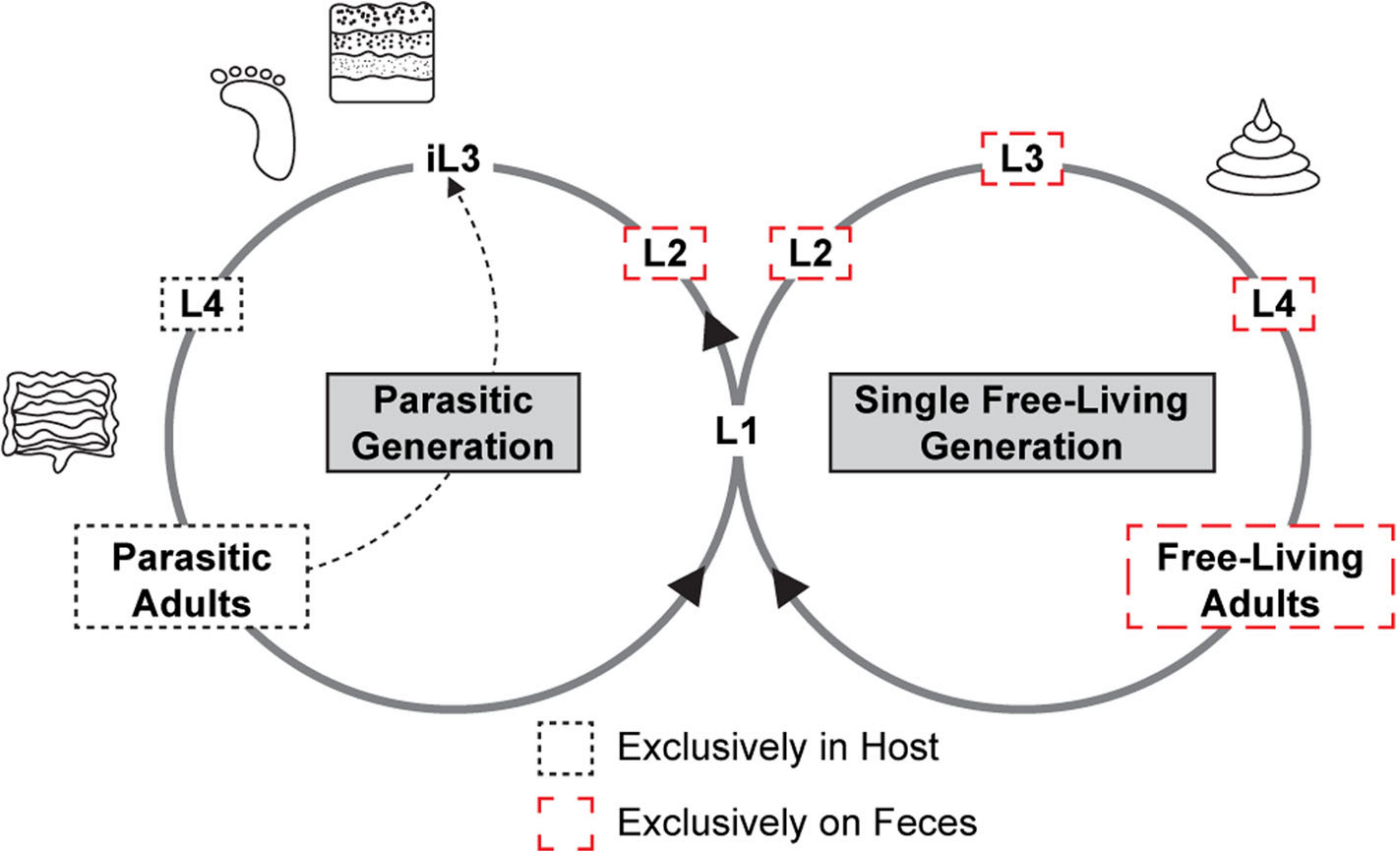
*Strongyloides* species encounter bacteria at specific points of their life cycle. The life cycles of the skin-penetrating gastrointestinal nematodes *S. stercoralis* and *S. ratti* consist of both a parasitic generation and a free-living generation. Developmentally arrested infective third-stage larvae (iL3s) search the environment for a host to infect. Once infection occurs via skin penetration, they develop into 4th stage larvae (L4s) and eventually into parasitic adults within the small intestine of the host. Parasitic adults reproduce asexually and their progeny exit the host in feces. Some of the population develops on feces through the 1st–4th larval stages (L1–L4) and then into free-living adults; the free-living adults reproduce sexually and their progeny develop into iL3s. The rest of the population develops through the 1st–2nd larval stages and then directly into iL3s. *S. stercoralis* uniquely can undergo autoinfection, whereby the progeny of the parasitic adults develop directly into iL3s within the host. Icons indicate environmental and host-associated bacterial niches the parasites encounter throughout their life cycle. The free-living generation and pre-iL3 life stages encounter fecal/gut bacteria; the iL3s encounter fecal/gut bacteria, host skin bacteria, and other environmental bacteria; and the parasitic life stages that exist inside the host may encounter host gut and fecal bacteria

Human-parasitic nematodes infect over a billion people worldwide and present a pressing issue for human health [1]. In low-resource countries, parasitic nematodes are one of the most common infectious agents of humans [2]. These parasites vary in their routes of infection; some infect by skin penetration, others by passive ingestion, and still others via intermediate hosts such as insect vectors [3]. *Strongyloides* is a genus of soil-dwelling, skin-penetrating gastrointestinal parasitic nematodes. This genus includes the human parasite *Strongyloides stercoralis* as well as the closely related rat parasite *Strongyloides ratti.* Current estimates put the global number of humans infected with *Strongyloides* at over 600 million [4]. Like many other parasitic nematodes, *Strongyloides* species develop through a number of free-living larval stages in the environment before becoming infective third-stage larvae (iL3s) [5]. However, *Strongyloides* species are unusual in that their life cycle also includes a species-specific number of free-living generations [6]. For example, *S. stercoralis* and *S. ratti* can cycle through a single free-living generation outside the host (Fig. 1) [3].

*Strongyloides* iL3s navigate their environment searching for a suitable host to infect and then penetrate host skin to establish an infection [3]. Once inside the host, the iL3s migrate through the host, ultimately ending up as parasitic adults in the small intestine [3]. All individuals of the parasitic generation are females that reproduce asexually by parthenogenesis [7]. The parasitic adults lay eggs in the intestine, and eggs or larvae exit the host in feces. On the feces, the progeny develop either directly into iL3s, or into free-living males and females that reproduce sexually. The free-living females lay eggs on the feces that develop into iL3s. The iL3s then migrate off of the feces and into the environmental soil to search for a host, thus completing the life cycle (Fig. 1) [3, 6].

*S. stercoralis* is found in warm climates throughout the world and accounts for the majority of human *Strongyloides* infections [8]. While these infections can be asymptomatic in healthy individuals, heavy infections can cause a range of symptoms that include gastrointestinal and respiratory distress [8]. *S. stercoralis* is particularly pathogenic due to its unique capacity for autoinfection, whereby the progeny of the parasitic generation develop directly into iL3s within the host [6].

Left untreated, hyperinfection can occur, which is characterized by an accelerated rate of autoinfection typically in response to a change in the immune status of the host. This can then result in disseminated strongyloidiasis, in which larvae migrate out of the small intestine. During disseminated strongyloidiasis, large numbers of autoinfective larvae invade the lungs, which contributes to the pulmonary symptoms associated with strongyloidiasis. Disseminated strongyloidiasis usually occurs in immunosuppressed individuals, such as those taking corticosteroids [9–12], and has a mortality rate approaching 100% if left untreated [12].

*S. ratti* is a close relative of *S. stercoralis* that also infects through skin penetration. It has a similar life cycle to *S. stercoralis* [3, 6, 13], but a more widespread geographic distribution [14]. *S. ratti* is distinct from *S. stercoralis* in that it naturally parasitizes brown rats (*Rattus norvegicus*) and is unable to cause autoinfection in the host [14]. *S. ratti* is a powerful comparative model for *S. stercoralis* because the two species are closely related genetically yet have distinct host ranges and geographical distributions.

Like many parasites, *Strongyloides* species encounter bacteria throughout their life cycle. The free-living life stages primarily encounter host fecal bacteria, the iL3s encounter both host-associated and environmental bacteria, and the parasitic stages primarily encounter host gut bacteria (Fig. 1). While some parasitic nematodes are known to form close associations with bacteria that are critical for parasite infectivity [15], the interactions of *Strongyloides* and other skin-penetrating nematodes with bacteria have not been examined. Whether these parasites associate with a specific microbiome, and whether they respond behaviorally and physiologically to host-associated and environmental bacteria, was not known.

Here, we investigated the interactions of *Strongyloides* species with bacteria. We first conducted a sequencing-based analysis of microbes associated with *S. stercoralis* and found that the free-living adults contained a microbiome consisting primarily of *Escherichia-Shigella*, *Bacteroides,* and *Lactobacillus* species, suggesting that they form specific associations with the bacteria on defecated host feces. We then asked how the free-living adults and iL3s of *Strongyloides* species, which engage in environmental navigation, respond to a diverse panel of host-associated and environmental bacteria. We found that *S. stercoralis* and *S. ratti* showed similar behavioral responses to the bacterial panel despite their different host ranges and geographical distributions. The responses of both species differed dramatically across life stages, with free-living adults responding broadly to most tested bacteria and iL3s responding specifically to a narrower range of environmental bacteria. Moreover, the short-term behavioral responses of *S. stercoralis* adults to bacteria differed from their longer-term responses, suggesting that *S. stercoralis* chemosensory behavior exhibits complex temporal dynamics. Finally, we found that the bacterium *Proteus mirabilis* reduced the reproductive success of *S. stercoralis* by decreasing egg hatching. Together, our results provide insight into the specific associations between *Strongyloides* species and bacteria, and identify a potential source of natural compounds that interfere with parasite development and may be useful for preventing or managing infections.

## Results

### *S. stercoralis* forms specific associations with host-associated bacteria

**Fig. 2:**
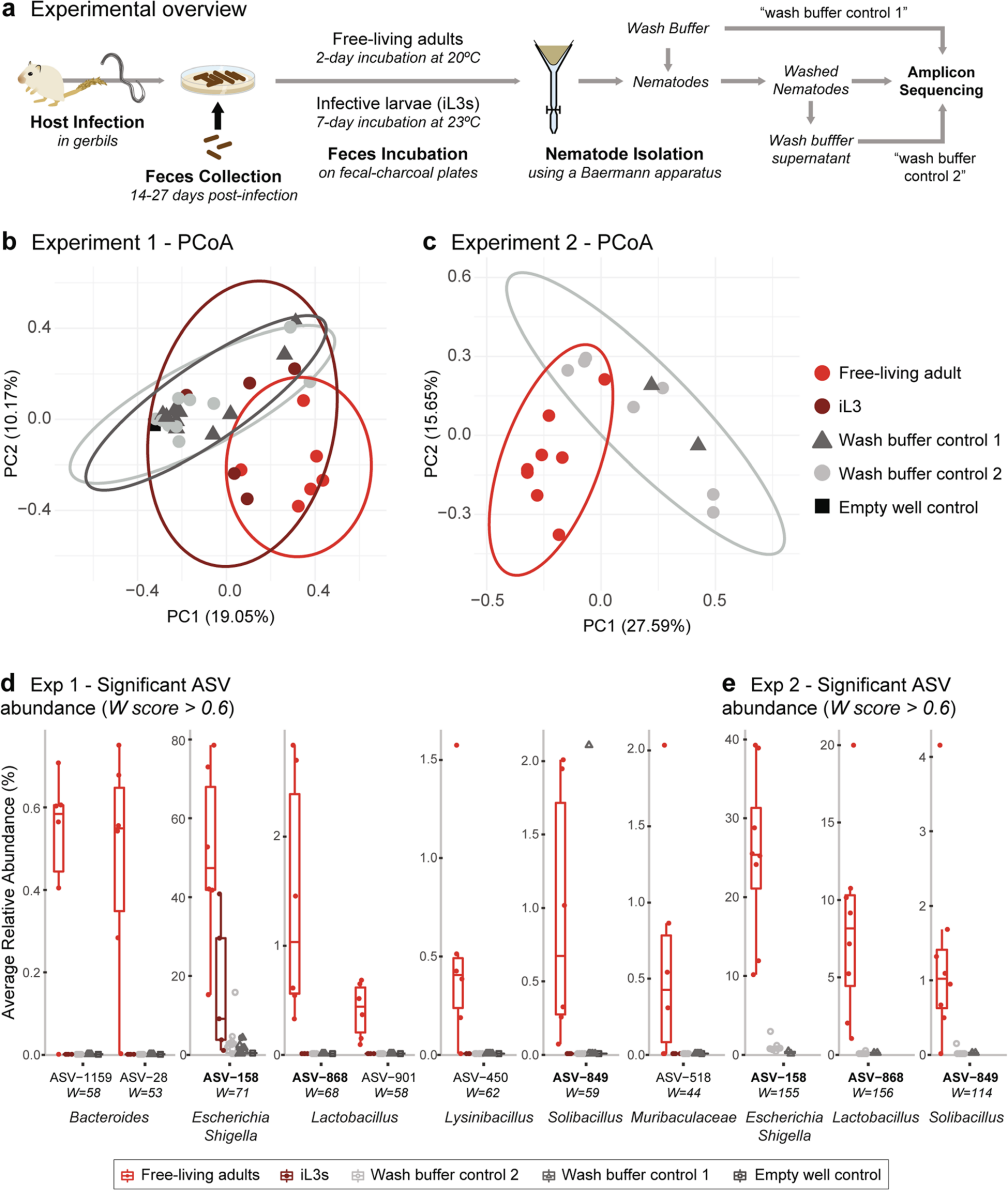
*S. stercoralis* free-living adults are associated with a specific microbiome. **a** Schematic of the experimental design. Gerbils were infected with *S. stercoralis* iL3s, and infested feces were harvested from the gerbils on days 14–27 post-infection. Collected feces were made into fecal-charcoal plates and incubated at either 20 °C for 2 days to obtain *S. stercoralis* free-living adults, or 23 °C for 7 days to obtain *S. stercoralis* iL3s. Nematodes were then isolated from the fecal-charcoal plates using a Baermann apparatus and washed in buffer before DNA extraction. Sequencing samples included DNA isolated from nematodes; wash buffer control 1, consisting of the buffer used to wash the nematodes prior to DNA extraction; and wash buffer control 2, consisting of the buffer supernatant after washing the nematodes. **b**–**e** Results from the amplicon sequencing analysis for Experiments 1 and 2. **b**, **c** Principal coordinates analysis (PCoA) of the different sample categories from Experiments 1 (**b**) and 2 (**c**). Free-living adult samples clustered separately from the other samples, suggesting a specific microbiome associated with *S. stercoralis* free-living adults. In contrast, some of the iL3 samples in Experiment 1 clustered with the control samples. In (**b**), an empty well control was also included. Ellipses representing the 95% confidence region for the sample groups with more than 3 samples were calculated using the ggplot2 and ellipse packages [73]. **d**, **e** Relative abundance of ASVs that showed a significant difference across sample categories in Experiments 1 (**d**) or 2 (**e**). Box plots are standard Tukey representations. ASVs with an ANCOM W score in the top 40% of all tested features were selected as significant; individual W scores are displayed below the ASV identifiers. ASV identifiers in bold are those that showed a significant enrichment in *S. stercoralis* free-living adults vs. controls in both sequencing experiments. ASVs in the genera *Escherichia-Shigella, Lactobacillus,* and *Solibacillus* were significantly enriched in the free-living adult samples relative to the other samples

The free-living nematode *Caenorhabditis elegans* tends to harbor specific bacteria in its gut and on its cuticle [16, 17]. However, the bacteria associated with skin-penetrating nematodes, including *Strongyloides* species, had not been characterized. To assess this, two independent sequencing experiments were performed where *S. stercoralis* free-living adults (and in one case iL3s) were isolated from fecal cultures using a Baermann apparatus [18] and 16S amplicon sequencing was used to characterize the nematode-associated microbes (Fig. 2). For these experiments, we included multiple levels of microbiome sequencing controls to facilitate robust interpretation of the results, including (i) wash buffer control 1, consisting of DNA isolated from the buffer that was used to wash the nematodes prior to nematode sample DNA extraction; (ii) wash buffer control 2, consisting of DNA isolated from the buffer supernatant after washing the nematodes; and (iii) an empty well control, consisting of an empty well that was processed along with the other samples during the amplicon sequencing workflow (Fig. 2a). Using principal coordinates analysis (PCoA), we found that the free-living adult samples clustered separately from the control samples in both independent experiments (Fig. 2b, c). Similarly, we found that the free-living adult samples had distinct microbiome profiles compared to controls (Additional files 1, 2, 3, 4 and 5: Fig. S1, S2, S3, S4 and S5). Several Amplicon Sequence Variants (ASVs) were significantly enriched in free-living adults relative to controls (Fig. 2d, e), including three individual ASVs identified in both experiments that belonged to the *Escherichia-Shigella* (ASV-158), *Lactobacillus* (ASV-868), and *Solibacillus* (ASV-849) genera. The most highly enriched ASV from the *Escherichia-Shigella* genera comprised between 30 and 60% of the nematode microbiomes between experiments (Fig. 2d, e, Additional file 1: Fig. S1). These results indicate that *S. stercoralis* free-living adults are associated with a robust microbiome.

For one of the experiments, we also performed a similar set of microbiome sequencing analyses on *S. stercoralis* iL3s. In contrast to the taxonomic composition of the free-living adult samples, the taxonomic composition of the iL3 samples was largely similar to that of the control samples, suggesting that iL3s may not have a distinct microbiome (Fig. 2b, Additional file 1: Fig. S1). The lack of an iL3 microbiome is consistent with the fact that the iL3 stage is non-feeding, and both the pharynx and intestine are remodeled in the iL3, a process that may involve expulsion of bacteria from the intestine [19, 20]. However, the same *Escherichia-Shigella* ASV that was observed in the free-living adult samples, ASV-158, was also observed in the iL3 samples, which suggests the potential for a specific association during the iL3 life stage as well. Together, our results suggest that *S. stercoralis* forms specific associations with bacteria at the free-living adult stage, some of which may extend to the iL3 stage.

### Behavioral responses of *S. stercoralis* to bacteria vary across life stages

**Fig. 3:**
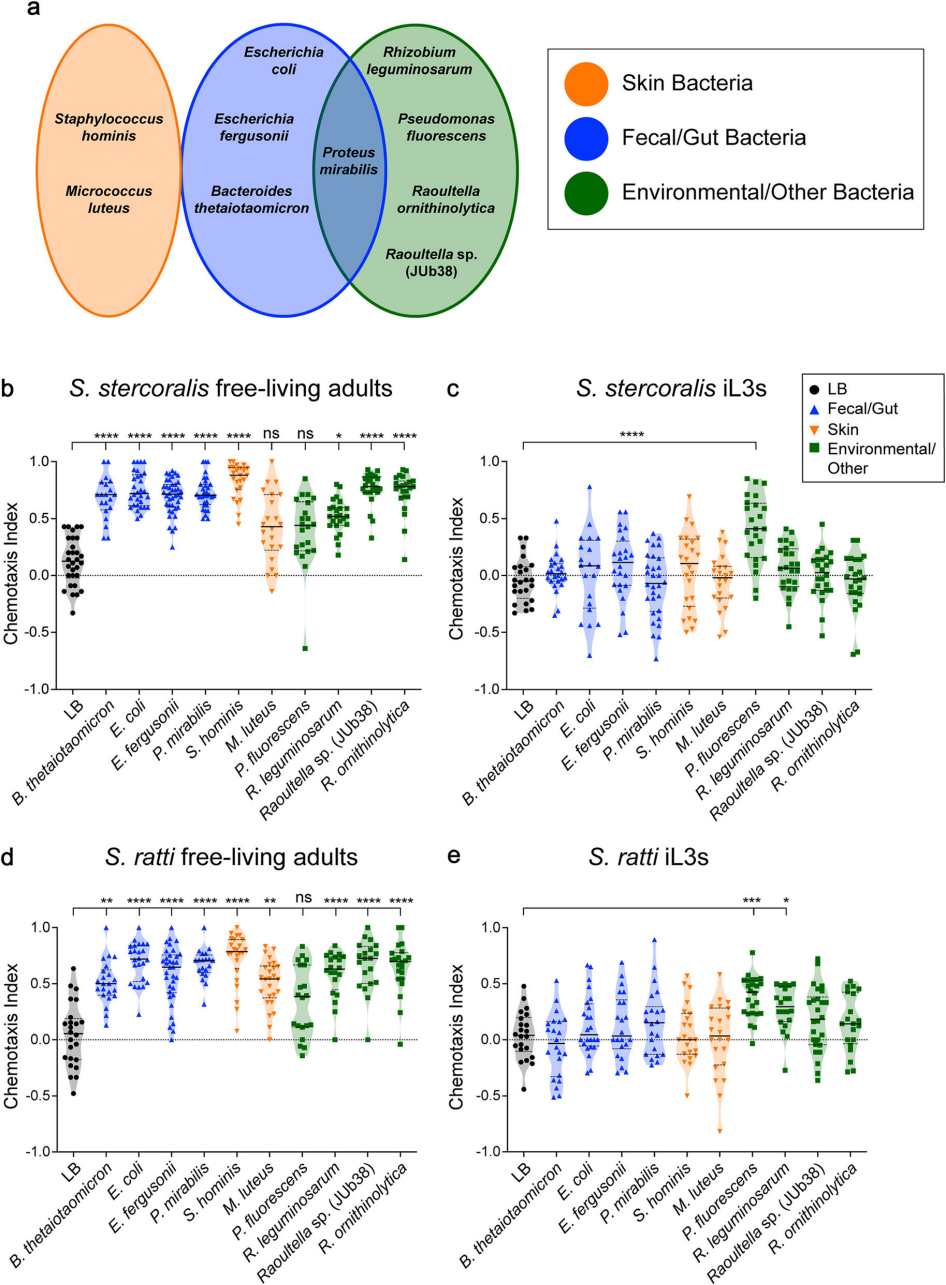
*Strongyloides* species display life-stage-specific bacterial preferences. **a** The bacterial panel used to examine the interactions of *Strongyloides* iL3s and free-living adults with bacteria. The bacteria are categorized according to the major environmental niches where they are likely to interact with *Strongyloides* species (Additional file 6: Table S1), although we note that some of the bacteria are also found more broadly in the environment. Categories (left to right): skin, fecal/gut, environmental/other. **b** *S. stercoralis* free-living adults were robustly attracted to most bacteria tested in a bacterial chemotaxis assay. *n* = 20–40 trials for each condition, with 75–150 worms per trial. *p< 0.05, ****p< 0.0001, ns = not significant, Kruskal-Wallis test with Dunn’s post-test. **c** *S. stercoralis* iL3s did not respond to most bacteria but were attracted to one of the environmental/other bacterial species tested. *n* = 20–30 trials for each condition, with 300-400 worms per trial. ****p< 0.0001, Kruskal-Wallis test with Dunn’s post-test. **d** *S. ratti* free-living adults were robustly attracted to most of the bacterial species tested in a bacterial chemotaxis assay. *n* = 20–34 trials for each condition, with 75–150 worms per trial. **p< 0.01, ****p< 0.0001, ns = not significant, Kruskal-Wallis test with Dunn’s post-test. **e** *S. ratti* iL3s did not respond to most bacteria but were attracted to two environmental/other bacterial species tested. *n* = 20–26 trials for each condition, with 300–400 worms per trial. *p< 0.05, ***p< 0.001, Kruskal-Wallis test with Dunn’s post-test. Each bacterial species was compared to the LB control; only significant differences are noted. Graphs show the chemotaxis indices for each trial (points), medians (solid lines), and interquartile ranges (dashed lines). Bacteria are color-coded according to the legend shown on the right

To assess the behavioral responses of *S. stercoralis* to bacteria, we measured the chemotactic behavior of both the free-living adult and iL3 life stages to a diverse panel of 10 different ethologically relevant bacteria (Fig. 3a). The bacterial species selected were broadly categorized into three groups depending on their typical environment: fecal/gut, skin, or environmental/other (Fig. 3a, Additional file 6: Table S1). *Strongyloides* species are likely to encounter bacteria from most of these environments during the course of their life cycle (Fig. 1). For example, the free-living adults encounter fecal bacteria since they reside on host feces; while the iL3s encounter fecal, environmental, and skin bacteria as they first migrate off of host feces and into the soil to host seek, and then later penetrate through host skin (Fig. 1) [21]. The two *Raoultella* bacterial strains used in the environmental/other category were originally isolated from rotting fruit, the type of environment containing *C. elegans* (rotting organic matter) [17]. They were tested to evaluate the response of *S. stercoralis* to bacteria from environments the parasites would not typically encounter. Responses to bacteria were measured using a bacterial chemotaxis assay in which nematodes were placed in the center of a plate containing a small lawn of bacteria on one side and a control consisting of the medium used for bacterial culturing on the other side, and allowed to migrate toward or away from the bacterial lawn (Additional file 7: Fig. S6a, left). Responses were quantified by calculating a chemotaxis index according to the number of worms in each region of the plate (Additional file 7: Fig. S6a, right). As expected, no behavioral preferences were observed in a series of control experiments containing only media on both sides of the plate (Additional file 7: Fig. S6b-e).

We first tested *S. stercoralis* free-living male and female adults and found that they were robustly attracted to most of the bacterial species tested (Fig. 3b). This includes the fecal/gut bacteria *Bacteroides thetaiotaomicron* (ATCC 29148), *Proteus mirabilis* (ATCC 29906), *Escherichia fergusonii* (ATCC 35469), and *Escherichia coli* (HB101); the skin bacterium *Staphylococcus hominis* (ATCC 27844); and the environmental/other bacteria *Rhizobium leguminosarum* (ATCC 14479), *Raoultella ornithinolytica* (JUb54), and a second *Raoultella* species (JUb38). Only two bacterial species tested, the skin bacterium *Micrococcus luteus* (ATCC 4698) and the environmental/other bacterium *Pseudomonas fluorescens* (ATCC 13525), were not significantly attractive (Fig. 3b). Thus, *S. stercoralis* free-living adults are broadly attracted to bacteria, including those from habitats they are unlikely to naturally encounter, such as rotting fruit.

In nature, *Strongyloides* free-living adults are thought to remain on feces and feed on fecal bacteria [8]. Given the robust attraction of the free-living adults to all three fecal/gut bacterial species tested—*E. coli, E. fergusonii,* and *P. mirabilis*—as well as the importance of fecal bacteria for the survival of the free-living generation (Fig. 1), we next asked if *S. stercoralis* adults prefer some fecal bacteria over others. We performed competition chemotaxis assays in which nematodes were allowed to choose between one of two fecal/gut bacterial species (Additional file 8: Fig. S7a). We found that *S. stercoralis* adults did not show a preference for *P. mirabilis* relative to *E. coli* or *E. fergusonii* (Additional file 8: Fig. S7b). However, they showed a slight preference for *E. coli* relative to *E. fergusonii* (Additional file 8: Fig. S7c). These results suggest that *S. stercoralis* free-living adults are capable of distinguishing among fecal/gut bacteria to some extent, but are generally strongly attracted to these bacteria.

Previous studies using individual odorants have shown that olfactory preferences in *Strongyloides* species can vary across life stages [22, 23]. To test whether bacterial preferences also vary across life stages, we next examined the responses of *S. stercoralis* iL3s, which are non-feeding and developmentally arrested [21], to the same bacterial panel. We found that in contrast to the free-living adults, the iL3s were neutral to most of the bacteria tested (Fig. 3c). The iL3s displayed significant attraction to only one bacterium tested, the environmental bacterium *P. fluorescens* (Fig. 3c). These data suggest that *S. stercoralis* shows life-stage-specific responses to bacteria. Free-living adults are robustly attracted to a broad range of bacteria, while iL3s are narrowly tuned to specific bacteria.

### *S. ratti* and *S. stercoralis* respond similarly to bacteria

Although *S. stercoralis* and *S. ratti* are closely related genetically and share similar life cycles, they infect distinct host species. Moreover, the bacterial composition of their hosts’ feces differs substantially; many of the core-OTUs found in human feces are absent or found at low abundance in rat feces [24, 25]. Thus, *S. stercoralis* and *S. ratti* are exposed to very different bacteria in their natural environments. In addition, the two species have different geographical ranges: *S. stercoralis* is found in warm regions throughout the world, while *S. ratti* is broadly distributed worldwide [14, 26]. This raises the question of whether *S. stercoralis* and *S. ratti* respond similarly to bacteria, or whether they exhibit species-specific responses to bacteria that reflect their unique environmental niches. To address this question, we first examined the chemotactic responses of *S. ratti* free-living adults to the bacterial panel (Fig. 3d). We found that *S. ratti* free-living adults were robustly attracted to most of the bacteria tested (Fig. 3d). *P. fluorescens* was the only bacterium that was not significantly attractive to *S. ratti* free-living adults (Fig. 3d). We then examined the behavioral responses of *S. ratti* iL3s to the bacterial panel and found that the iL3s were neutral in response to most bacteria tested (Fig. 3e). The only significantly attractive bacteria in the panel were the two environmental/other bacteria, *P. fluorescens* and *R. leguminosarum* (Fig. 3e).

A comparison of *S. ratti* and *S. stercoralis* free-living adults revealed no significant differences in behavioral responses to the bacteria (Additional file 9: Fig. S8a), and a comparison of *S. ratti* and *S. stercoralis* iL3s revealed only a minor difference in the response to *P. mirabilis* (Additional file 9: Fig. S8b). Thus, the life-stage-dependent bacterial preferences of *S. ratti* closely match those of *S. stercoralis*. Interestingly, the environmental/other bacterium *P. fluorescens* was not significantly attractive to adults of either nematode species, yet it was the only bacterium significantly attractive to iL3s of both species (Fig. 3b–e). This bacterium is commonly found in association with soil, plants, and water [27, 28]. The overall similarity in the bacterial preferences of *S. ratti* and *S. stercoralis* suggests that these species do not show highly specialized behavioral responses to bacteria that reflect their species-specific fecal niches. Instead, the two species show very similar behavioral responses that may reflect their similar life cycles and/or genetic relatedness.

### *S. stercoralis* free-living adults display dynamic responses to bacteria

**Fig. 4:**
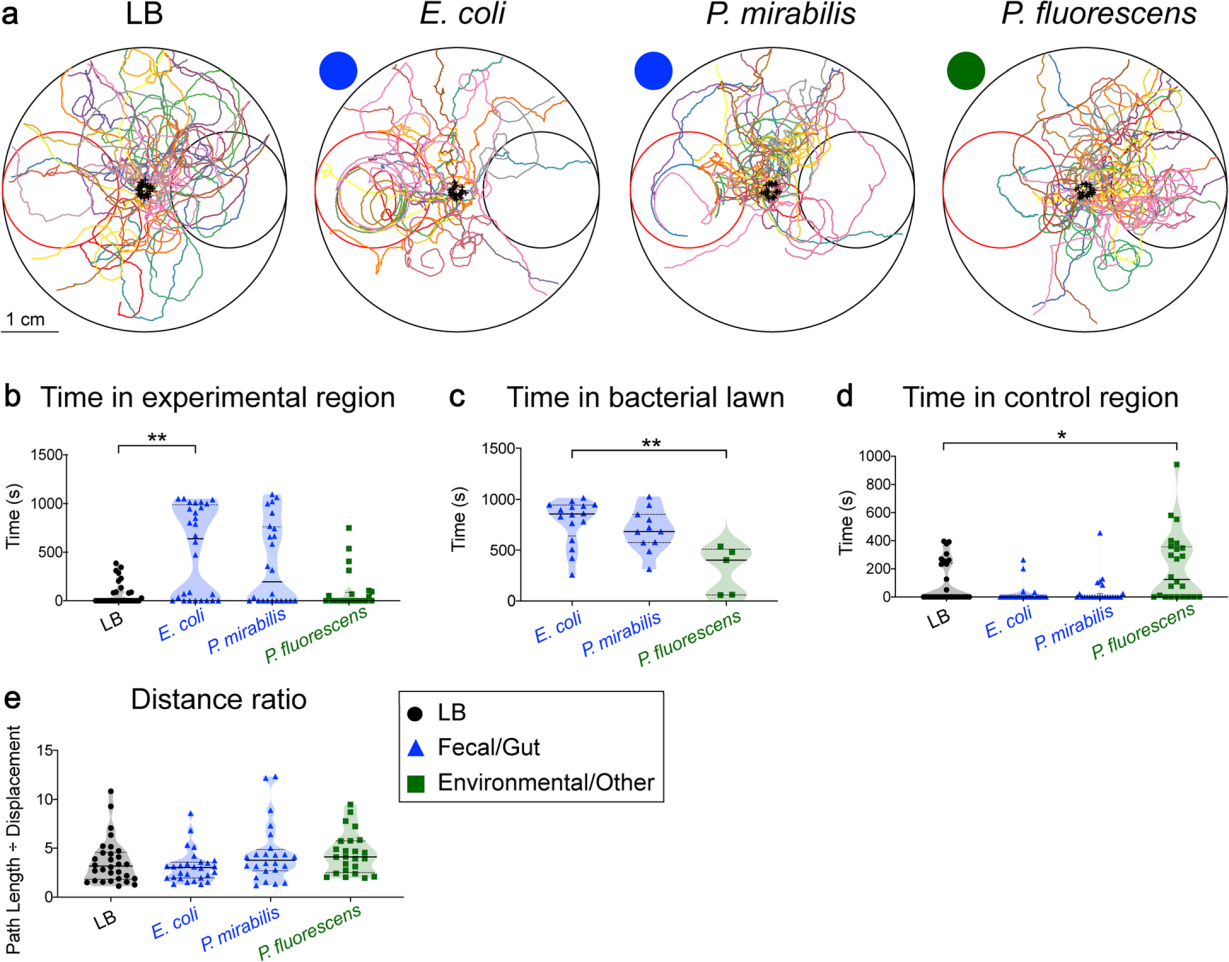
*S. stercoralis* free-living adults show distinct short-term behavioral responses to bacteria. **a** Overlaid tracks of individual *S. stercoralis* free-living adults in a single-worm bacterial chemotaxis assay. Worms were tracked for 20 min or until they left the assay arena. Black crosses represent starting points. For each assay arena, the red circle (left) depicts the experimental zone containing bacteria and the black circle (right) depicts the control zone containing the media control. In the case of the LB control assay, both the experimental zone and the control zone contained LB media. **b** Worms spent more time in the experimental zone when the experimental zone contained *E. coli*. **p< 0.01, Kruskal-Wallis test with Dunn’s post-test. The only significant difference is noted. **c** Worms spent more time in the *E. coli* lawn than they did in the *P. fluorescens* lawn. Only animals that reached a bacterial lawn were included in this analysis. **d** Worms spent more time in the control zone when the experimental zone contained *P. fluorescens*. *p< 0.05, Kruskal-Wallis test with Dunn’s post-test. The only significant difference is noted. **e** Worms displayed similar navigational patterns regardless of the bacteria present, as quantified by the distance ratio (total pathlength ÷ linear displacement from the initial point to the final point). No significant differences were detected (Kruskal-Wallis test). *n* = 24–30 worms for each condition. Graphs show medians (solid lines) and interquartile ranges (dashed lines). All statistical comparisons are relative to the LB control assay

Population chemotaxis assays are strong indicators of overall chemosensory preferences, but do not provide insight into either the immediate responses of the nematodes to the chemosensory cues or the navigational strategies employed during chemotaxis. To address these points, we used single-worm tracking assays in which a single *S. stercoralis* free-living adult was placed in the center of a plate containing a small circle of bacteria on one side of the plate (the “experimental region”) and a small circle of an LB media control on the opposite side (the “control region”) (Additional file 10: Fig. S9). An older, gravid female was assayed to control for any mate-seeking effects. The worm was then tracked with video recording over a maximum of 20 min (Additional file 10: Fig. S9). We tracked *S. stercoralis* adults as they navigated across plates containing either *E. coli*, *P. mirabilis*, or *P. fluorescens* (Fig. 4a). We compared responses to these bacteria to responses to control plates containing LB media on both sides of the plate. As expected, the trajectories of worms on the LB control plates appeared to be relatively evenly distributed across the plate (Fig. 4a).

A comparison of responses to *E. coli*, *P. mirabilis*, *P. fluorescens*, and the LB control revealed that *S. stercoralis* adults were more strongly attracted to *E. coli* than *P. mirabilis* in the short-term tracking assay (Fig. 4a). Adults spent significantly more time in the experimental region on plates containing *E. coli* than plates containing only the LB control (Fig. 4b). In contrast, the time spent in the experimental region on plates containing the other bacteria did not differ significantly from the time spent in the experimental region on plates containing only the LB control (Fig. 4b). To assess how long the bacteria retained the adults after they reached it, we compared the dwell time in the bacterial lawns across conditions. Adults spent significantly more time in the *E. coli* lawn relative to the *P. fluorescens* lawn (Fig. 4c). To control for the possibility that the bacterial lawns differentially impeded worm speed, the median crawling speeds in the bacterial lawns were quantified. There were no significant differences in nematode crawling speeds on the different bacterial lawns (Additional file 11: Fig. S10). Together, these results suggest that *E. coli* is more attractive initially than *P. mirabilis*, even though in the longer-term population chemotaxis assays, worms eventually accumulate in both bacterial lawns in similar numbers. Additionally, our data suggest that *E. coli* retains the adults longer than *P. fluorescens* (Fig. 4c). Single-worm tracking analysis also revealed that worms on plates containing *P. fluorescens* spent more time in the control region than worms on plates containing only the LB control (Fig. 4d). Thus, *S. stercoralis* adults show an initial repulsion from *P. fluorescens*, even though *P. fluorescens* appears to be neutral in the longer-term population assay (Fig. 3b).

We then investigated the navigational strategies used during bacterial chemotaxis. We calculated the distance ratio (total path length ÷ displacement) to quantify the nonlinearity of the tracks, and found no significant differences in distance ratio across conditions (Fig. 4e). Together, these results suggest that *S. stercoralis* free-living adults are capable of distinguishing among different bacterial species and display distinct short-term bacterial preferences that may differ from their long-term preferences. However, similar navigational strategies appear to be employed regardless of whether the bacteria are attractive or repulsive.

### Growth on *P. mirabilis* decreases egg hatching in *S. stercoralis*

**Fig. 5:**
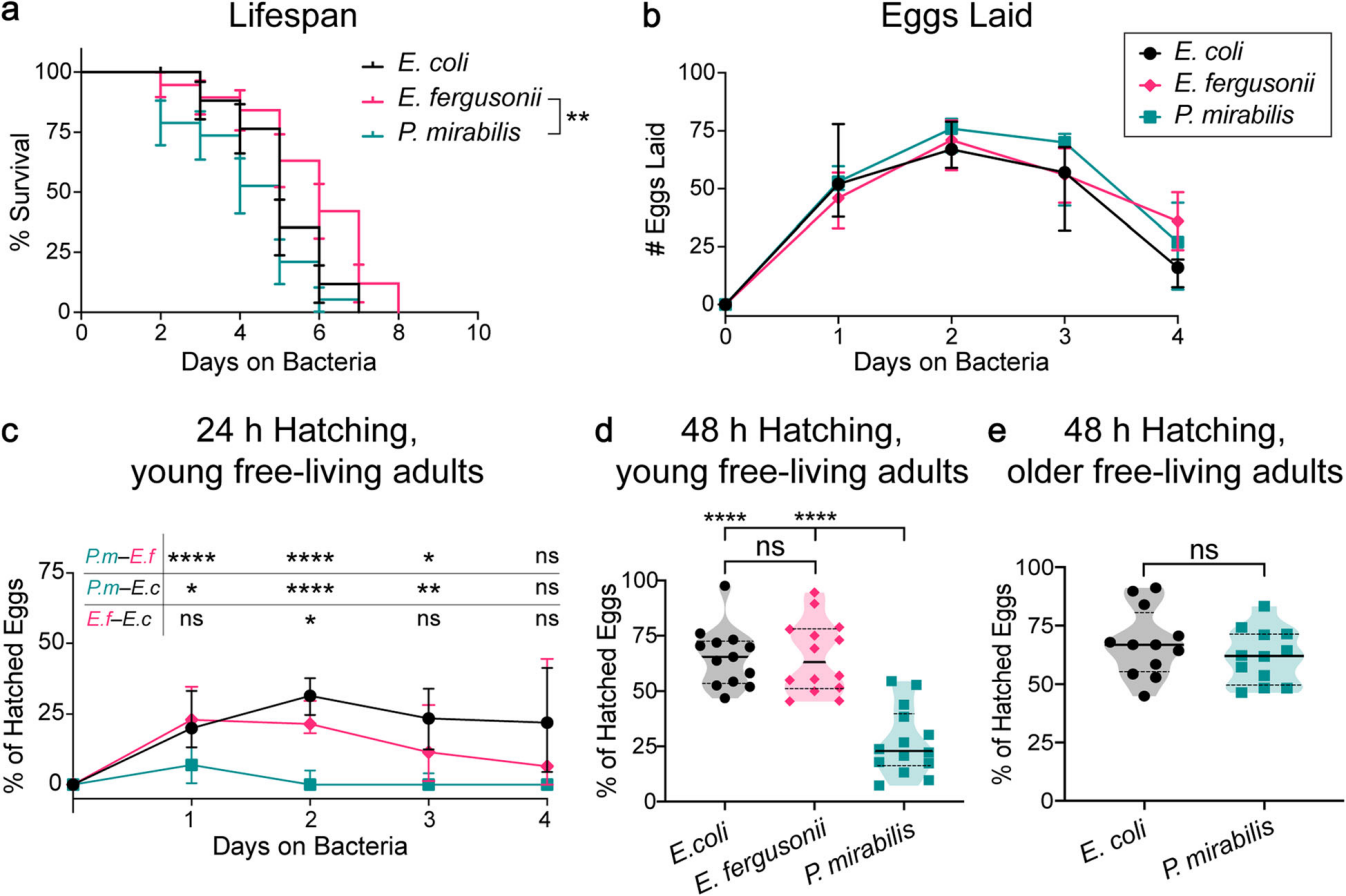
Fecal/gut bacteria influence *S. stercoralis* physiology. **a** Survival of *S. stercoralis* free-living adult females cultured on either *E. coli, E. fergusonii,* or *P. mirabilis.* Worms were placed on plates containing the indicated bacteria as young adults, and percent survival was monitored daily. *n* = 19 worms for each condition. **p< 0.01, log-rank test with Bonferroni post-test comparing each condition to every other condition. The only significant difference is noted. Error bars show standard error. **b** Culturing *S. stercoralis* free-living adults on different fecal bacteria did not affect the number of eggs laid per day. No significant effect of bacteria was detected (two-way repeated measures ANOVA). *n* = 13–17 worms for each condition. Medians and interquartile ranges are shown. **c** Culturing *S. stercoralis* free-living adults on *P. mirabilis* resulted in fewer hatched eggs per day. Graph shows the percentage of eggs that hatched every 24 h after free-living young adult females were allowed to mate on the indicated bacteria. *p< 0.05, **p< 0.01, ****p< 0.0001, ns = not significant, two-way repeated measures ANOVA with Tukey’s post-test. Statistical significance for each comparison is noted above the graph. *n* = 12-16 worms for each condition. Medians and interquartile ranges are shown. **d** *P. mirabilis* decreased egg hatching. Graph shows the percentage of hatched eggs 48 h after free-living young adult females were allowed to mate on the bacteria. ****p< 0.0001, ns = not significant, Brown-Forsythe and Welch ANOVA with Dunnett’s T3 post-test comparing each condition to every other condition. *n* = 13–14 worms for each condition. Medians (solid lines) and interquartile ranges (dashed lines) are shown. **e** There was no significant difference in the percentage of hatched eggs after 48 h when older, gravid free-living adult females were placed on *P. mirabilis* (unpaired, two-tailed Welch’s *t* test). *n* = 12 worms for both conditions. Medians (solid lines) and interquartile ranges (dashed lines) are shown

Although the free-living life stages of *S. stercoralis* can be maintained on *E. coli* in a laboratory setting, their natural habitat is host feces. This raises the question of how their lifespan and physiology is influenced by other fecal bacteria. To begin to address this question, *S. stercoralis* free-living young adult females were isolated from feces and cultured on plates containing a lawn of either *E. coli*, *E. fergusonii*, or *P. mirabilis* for the extent of their lifespan. The nematodes were passaged daily to fresh bacterial plates, and their progeny were counted daily (Additional file 12: Fig. S11a). We found only minor differences in the lifespans of worms cultured on the different bacterial plates, with worms cultured on *E. fergusonii* showing slightly longer lifespans than worms cultured on *P. mirabilis* (Fig. 5a). We did not find any significant differences in the number of eggs laid on the different bacteria (Fig. 5b). However, when we examined the viability of the eggs laid on the different bacteria, we found that significantly fewer eggs hatched after 24 h on *P. mirabilis* than on the other bacteria (Fig. 5c). These results suggest that *P. mirabilis* is detrimental to the reproduction of *S. stercoralis* free-living adults.

The reduced hatching of *S. stercoralis* eggs after 24 h when raised in *P. mirabilis* could reflect either reduced egg viability or delayed egg hatching, such that the eggs take longer than 24 h to hatch. To distinguish between these possibilities, we examined egg hatching after 48 h instead of 24 h to give the eggs additional time to hatch (Additional file 12: Fig. S11b). Similar to the results with the 24 h assay, we found that *P. mirabilis* significantly reduced the percentage of hatched eggs after 48 h relative to *E. coli* and *E. fergusonii* (Fig. 5d). No significant difference in egg hatching between worms cultured on *E. coli* versus *E. fergusonii* was observed (Fig. 5d). These results suggest that *P. mirabilis* primarily reduces egg viability.

The reduced egg viability of *S. stercoralis* females grown on *P. mirabilis* could be due to an effect of *P. mirabilis* on either the young adult female or the laid egg. To distinguish between these possibilities, we examined egg hatching after 48 h using older, gravid adult females instead of young adult females (Additional file 12: Fig. S11c). The older females had already mated and developed eggs in the absence of *P. mirabilis,* and thus any effect of *P. mirabilis* on *S. stercoralis* would be primarily restricted to egg laying or to the development of the eggs once laid. In this assay, no significant difference in egg hatching was observed for worms on *P. mirabilis* versus *E. coli* (Fig. 5e). Thus, *P. mirabilis* appears to inhibit egg hatching by affecting the young free-living adult female directly.

To investigate how *P. mirabilis* inhibits egg hatching, we asked if soluble or volatile factors produced by *P. mirabilis* were sufficient to inhibit *S. stercoralis* egg hatching. To test whether soluble factors produced by *P. mirabilis* can inhibit egg hatching, young adults were plated on a lawn of *E. coli* to which filtered bacterial supernatant from either *E. coli* or *P. mirabilis* was added. Egg hatching was then assessed over 48 h. There was no significant difference in egg hatching on plates containing *E. coli* vs. *P. mirabilis* supernatant (Additional file 13: Fig. S12a). To test whether volatile factors produced by *P. mirabilis* can inhibit egg hatching, young adults were plated on a lawn of *E. coli*, and a second plate containing a lawn of either *E. coli* or *P. mirabilis* was then affixed over the plate containing the worms. With this setup, worms were exposed to volatiles from either *E. coli* or *P. mirabilis* but encountered only *E. coli* as a nutrient source. We found that there was no significant difference in egg hatching in the presence of *E. coli* vs. *P. mirabilis* volatiles (Additional file 13: Fig. S12b). These results suggest that intact bacterial cells of *P. mirabilis* are likely necessary to inhibit egg hatching.

### *P. mirabilis* interferes with *S. stercoralis* early egg development

**Fig. 6:**
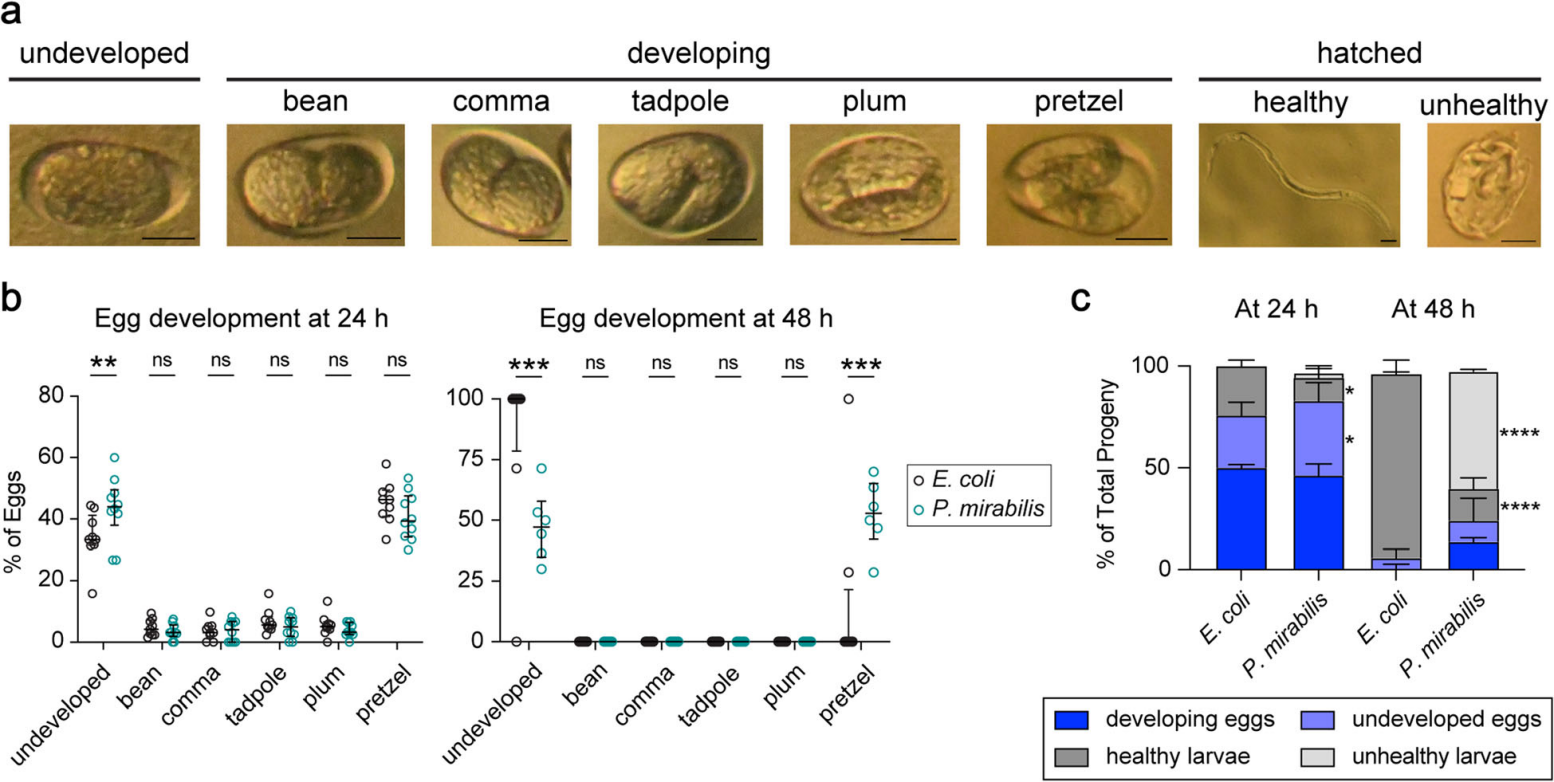
*P. mirabilis* impairs both egg and larval development in *S. stercoralis*. **a** Representative images of *S. stercoralis* embryonic development, a healthy pre-infective larva, and an unhealthy pre-infective larva. Egg development was classified into the categories indicated based on the classifications used for *C. elegans* [29]. Scale bars = 20 μm. **b** The percentage of unhatched eggs that fell into each category of embryonic development for worms cultured on either *P. mirabilis* or *E. coli*. For each experiment, 1 female and 3 males were cultured on the bacterial lawn for 24 h, after which the worms were removed from the plate and the percentage of eggs in each category was scored. The percentage of eggs in each category was then scored again after 48 h. After 24 h, more undeveloped eggs were observed on *P. mirabilis* relative to *E. coli*, suggesting that *P. mirabilis* interferes with the earliest stages of egg development (left). **p< 0.01, ns = not significant, two-way ANOVA with Sidak’s multiple comparisons post-test. *n* = 9–10 trials for each condition. After 48 h, nearly all of the eggs on the *E. coli* plates had hatched; the remaining eggs were almost all undeveloped. In contrast, approximately half of the eggs on *P. mirabilis* were at the pretzel stage of development while the other half were undeveloped, indicating that culturing on *P. mirabilis* delays or prevents egg hatching in some eggs that have initiated embryonic development (right). ***p< 0.001, ns = not significant, two-way ANOVA with Sidak’s multiple comparisons post-test. *n* = 6-10 trials for each condition. **c.** The percentage of the total progeny consisting of undeveloped eggs, developing eggs, healthy larvae, or unhealthy larvae from the experiment described in (**b**) after 24 h (left) or 48 h (right). Culturing on *P. mirabilis* resulted in an increased proportion of eggs that failed to develop and of the worms that hatched, an increased number of unhealthy larvae. Larvae that were classified as unhealthy appeared non-motile and generally remained in a curled position. *p< 0.05, ****p< 0.0001, two-way ANOVA with Tukey’s multiple comparisons test. Comparisons are between the same category and time point; only significant differences are shown. *n* = 7–10 trials for each condition. Median percentages are represented, and bars represent interquartile ranges. Data in (**b**) and (**c**) are from the same experiments

To further characterize the deleterious effects of *P. mirabilis* on *S. stercoralis* embryonic development, we examined the developmental stages of eggs laid on lawns of *P. mirabilis*. For this experiment, young adult males and females were placed on lawns of *P. mirabilis* for up to 48 h, and eggs and young larvae were morphologically categorized after 24 h and 48 h (Fig. 6a) [29]. Of the unhatched eggs at the 24 h timepoint, we found a higher proportion of eggs to be undeveloped on *P. mirabilis* plates relative to *E. coli* plates (Fig. 6b). Subcategorizing the developing eggs revealed that most of the developing eggs were at the final pretzel stage of development by 24 h (Fig. 6b, left). A similar categorical distribution of developing eggs was observed on *E. coli* vs. *P. mirabilis* plates after 24 h. By 48 h, virtually all eggs left unhatched were either undeveloped or in the final pretzel stage (Fig. 6b, right). At 48 h on *E. coli* plates, the majority of the few remaining unhatched eggs were undeveloped and very few were in the pretzel stage. In contrast, of the unhatched eggs on *P. mirabilis* plates at the same timepoint, approximately half were in the undeveloped stage and half were in the pretzel stage. These results further suggest that *P. mirabilis* may prevent or delay egg hatching in a subset of eggs that initiate embryonic development. Of the larvae that did hatch on *P. mirabilis* plates by 48 h, a majority were malformed and morphologically distinct from healthy larvae; these larvae were unmoving and often appeared tightly curled in on themselves (Fig. 6a, c). These results suggest that in the presence of *P. mirabilis,* fewer *S. stercoralis* eggs initiate embryogenesis.

*P. mirabilis* could reduce the number of eggs that undergo embryogenesis either directly by interfering with egg development, or indirectly by interfering with *S. stercoralis* mating behavior, which in turn could result in the females laying more unfertilized eggs. To distinguish between these possibilities, we plated lone young adult females on *P. mirabilis* plates for 24 h and then determined the percentage of eggs that had hatched at 24 and 48 h. We found that hatching was decreased on *P. mirabilis* at both 24 h and 48 h despite the absence of males (Additional file 14: Fig. S13a). Moreover, a population of persisting developing eggs and malformed larvae on *P. mirabilis* plates was also seen in these conditions at 48 h (Additional file 14: Fig. S13b). These results suggest that the presence of males is not necessary to recapitulate the impaired hatching phenotype, and therefore that *P. mirabilis* does not exert its effect on egg viability by interfering with the mating process. Finally, we assessed egg hatching at 24 h after a shorter egg-laying period of 12 h, and found that egg hatching was similar on *E. coli* vs. *P. mirabilis* plates under these conditions (Additional file 14: Fig. S13c). Thus, the detrimental effects of *P. mirabilis* are specific to the later-laid eggs.

Taken together, our results suggest that *P. mirabilis* causes reduced egg viability through a detrimental effect on the process of early egg development inside the young adult female. The finding that *P. mirabilis* is detrimental for the viability of the human parasite *S. stercoralis* raises the possibility that *P. mirabilis* could be useful in the development of new compounds for nematode control.

## Discussion

We have found that *S. stercoralis* free-living adults are associated with a specific microbiome (Fig. 2). The *S. stercoralis* microbiome appears to be relatively simple in composition: it is dominated by a single bacterium in the *Escherichia-Shigella* genus, but also includes at least two other bacteria in lower abundance, one in the genus *Lactobacillus* and the other in the genus *Solibacillus* (Fig. 2, Additional files 1, 2, 3, 4 and 5: Fig. S1, S2, S3, S4 and S5). In contrast, the *C. elegans* microbiome is enriched in specific genera such as *Pseudomonas*, *Stenotrophomonas*, *Ochrobactrum*, *Sphingomonas*, and those of the *Enterobacteriaceae* family [30], consistent with its different ecological niche. The bacteria we identified from *S. stercoralis* are likely to colonize the nematode gut given the consistency of their association in samples, but it is possible that they may adhere to the nematode cuticle; further studies will be necessary to distinguish between these possibilities.

Previous studies have identified *Lactobacillus* in the feces of Mongolian gerbils [31], indicating that the rodent is naturally associated with this genus. Although *Lactobacillus* bacteria are traditionally classified as oxygen-tolerant anaerobes, many species can grow under aerobic conditions [32–34]. Thus, it is unlikely that our experimental settings selected for *Lactobacillus* due to differences in oxygen concentration between the nematode and the environment. Interestingly, *Solibacillus* bacteria form spores, a dormant life stage that enables bacterial survival under unfavorable conditions [35–38]. Spore formation has been implicated in host-to-host transmission, since the inability of hosts to digest spores can lead to enhanced intestinal colonization [35, 39]. Thus, spore formation may be important for the ability of *Solibacillus* to colonize *S. stercoralis*. However, additional work will be needed to better understand the mechanisms that promote the association of *Lactobacillus* and *Solibacillus* with *S. stercoralis*.

Other intestinal parasites such as the murine whipworm *Trichuris muris* have been shown to harbor a gut microbiome that includes related *Enterobacteriaceae* and *Bacteroides* species [40]. *Lactobacillus* species in the host duodenum are also positively correlated with susceptibility to infection with some gastrointestinal nematodes, such as the passively ingested murine parasite *Heligmosomoides polygyrus* [41] and *T. muris* [42]. In addition, some *Lactobacillus* species induce hatching of *T. muris* eggs [43]. Thus, our results raise the possibility of a broader association between some microbial taxa and mammalian-parasitic nematodes.

The role of the *S. stercoralis* microbiome in shaping the interactions of *S. stercoralis* with its human hosts remains unclear. A number of gastrointestinal nematodes, including *S. stercoralis*, alter the bacterial composition of their host’s microbiome [44–49]. This effect is generally thought to result from the immune response to the parasitic infection rather than from bacteria transported into the host from the parasite [44, 45, 48]. In the case of *T. muris*, which infects when its eggs are ingested, the eggs are sterile and the microbiome associated with subsequent life stages of *T. muris* is acquired from the host intestine [15, 40]. In contrast, the ruminant parasite *H. contortus*, which infects when third-stage larvae are ingested, is associated with bacterial endosymbionts that are not found in the host abomasum or feces [50]. These endosymbionts are associated with *H. contortus* across life stages and vertically transmitted [50]. Thus, at least some parasitic nematodes are capable of transporting bacteria into their hosts during infection. Our results suggest that *S. stercoralis* iL3s may be associated with a bacterium in the genus *Escherichia-Shigella* (Fig. 2, Additional files 1, 2 and 3: Fig. S1, S2 and S3), raising the possibility that some iL3s transport bacteria into the host. However, it is also possible that the host microbiome changes associated with *S. stercoralis* infection result from the immune response to infection rather than from colonization with bacteria introduced by the iL3s.

At the behavioral level, both *S. stercoralis* and *S. ratti* showed life-stage-specific responses to bacteria: the free-living adults were robustly attracted to most bacteria tested, whereas iL3s were only attracted to specific bacteria commonly found in extra-host environments (Fig. 3). The robust attraction displayed by the free-living adults likely represents a drive toward nutrients, since the adults feed on host fecal bacteria [8]. Interestingly, we found that the free-living adults were attracted not only to fecal/gut bacteria, but also to those in the skin and environmental/other categories (Fig. 3, Additional file 6: Table S1). Thus, the free-living adults are broadly attracted to many bacteria, including bacteria they would not normally encounter in nature. Attraction to bacteria may be a relatively non-specific response employed by adults that serves to retain them on feces, which is a bacteria-rich food source. Notably, free-living adults were not robustly attracted to *P. fluorescens* and *R. leguminosarum*, which are commonly found in soil environments. *S. stercoralis* free-living adults were not attracted to *P. fluorescens* and were only moderately attracted to *R. leguminosarum* (Fig. 3b), while *S. ratti* free-living adults were not attracted to *P. fluorescens* but were attracted to *R. leguminosarum* (Fig. 3d). The reduced attraction to these bacteria exhibited by both species may be another mechanism that contributes to the tendency of adults to remain on feces rather than migrate into the surrounding soil environment.

In contrast to the free-living adults, the developmentally arrested iL3s migrate off feces and into the soil to search for hosts to infect. This change in behavior is reflected in a corresponding change in bacterial preferences. The iL3s of both species are neutral in response to most bacteria but are specifically attracted to either one (in the case of *S. stercoralis*) or two (in the case of *S. ratti*) of the tested bacteria that may be found in the surrounding soil environment (Fig. 3c, e). Thus, the behavioral responses of *Strongyloides* species to bacteria differ dramatically across life stages. Our results are consistent with previous studies showing that *S. stercoralis* and *S. ratti* adults are attracted to host fecal odor, while iL3s are neutral [22, 23]. The lack of attraction of iL3s to fecal/gut bacteria, combined with their attraction to environmental bacteria, may facilitate the movement of iL3s off of host feces and into the surrounding soil, where they then engage in host seeking.

Overall, *S. stercoralis* and *S. ratti* showed similar responses to bacteria at both the free-living adult and iL3 life stages (Additional file 9: Fig. S8) despite their differences in host range. These results suggest that the responses to the bacteria tested are not likely to be important drivers of species-specific parasite-host interactions. However, we note that the *S. stercoralis* free-living adults and iL3s tested here were cultured in the lab on gerbil feces rather than human feces; future studies will be necessary to examine the bacterial preferences of wild isolates of *S. stercoralis* cultured on human feces. While *S. stercoralis* and *S. ratti* appear to respond similarly to bacteria, they respond very differently to a diverse array of host-emitted odorants [22, 23]. Moreover, a comparison of iL3 olfactory preferences across species revealed that the olfactory responses of iL3s were more similar for nematode species that infect the same host species [22, 23]. These results suggest that olfaction contributes to the ability of different iL3s to target distinct host species. It is possible that the similar responses of *S. stercoralis* and *S. ratti* to the tested bacteria serve the more general role of maintaining the free-living life stages on bacterial food sources and promoting migration into the soil at the iL3 stage. However, *S. stercoralis* and *S. ratti* may respond in a species-specific manner to host-specific bacteria that have not been tested.

*S. stercoralis* iL3s are attracted to human sweat and serum, while *S. ratti* iL3s are attracted to mammalian serum [51, 52]. Moreover, *S. stercoralis* iL3s are attracted to skin-enriched odorants [22, 23]. Despite this, we observed no attraction to the human skin bacteria tested (Fig. 3c, e). It is possible that other as-yet-untested microbes found on mammalian skin are attractive to iL3s. However, it is also possible that a complex host skin microbiome consisting of multiple bacterial species is necessary to attract iL3s to host skin. Future studies examining the responses to a larger panel of skin bacteria or co-cultures of bacteria would shed light on this question.

The results of the single-worm tracking assays suggest that behavioral responses to bacteria can be temporally dynamic. While *P. fluorescens* was neutral for *S. stercoralis* free-living adults in the long-term assay, short-term tracking revealed it to be initially repulsive (Fig. 4a, d). It is possible that adults initially sense *P. fluorescens* as noxious or unfavorable, but over time, they acclimate to it if it is the only potential food source available. Moreover, *P. mirabilis* was not as strongly attractive in the short-term tracking assay as it was in the long-term population assay (Figs. 3b and 4a, b), highlighting the importance of exposure time and nutrient availability in driving these responses. Thus, while *S. stercoralis* free-living adults show broad, relatively non-specific attraction to bacteria (Fig. 3b), our results from short-term tracking assays (Fig. 4) and bacterial competition assays (Additional file 8: Fig. S7) suggest that they are able to discriminate between different bacterial species and respond in slightly different ways. The distance ratio did not differ significantly regardless of the bacterial species (Fig. 4e), suggesting that generally similar navigational strategies may be employed in both attractive and repulsive behavioral responses to bacteria.

Given the bacteria-rich environments of the human gut and feces, where *S. stercoralis* reproduces, human-associated bacteria are likely to play an important role in regulating parasite propagation. In the case of the mouse parasite *Strongyloides venezuelensis*, administration of the probiotic *Bifidobacterium animalis* to infected mice significantly reduces egg output [53]. Similarly, altering the bacterial culture conditions of *S. ratti* results in changes in egg laying and hatching [54]. Our results demonstrate that culturing *S. stercoralis* free-living young adults on *P. mirabilis* results in a dramatically reduced rate of egg hatching (Fig. 5c, d). The bacterium exerts its effect on the nematodes by interfering with early egg development, since eggs from older, gravid females are unaffected by the bacterium (Fig. 5e). Moreover, young adult females exposed to the bacterium lay eggs that are less likely to initiate embryogenesis (Fig. 6). *P. mirabilis* also reduces the brood size of *C. elegans* [55], but whether the mechanisms underlying these effects in *S. stercoralis* and *C. elegans* are similar remains unclear. In future studies, it will be interesting to extend these studies to *S. stercoralis* parasitic adults, since the free-living versus parasitic life stages of *S. stercoralis* may interact differently with host-associated bacteria.

Interestingly, *S. stercoralis* adults are robustly attracted to *P. mirabilis* despite its negative effect on reproduction (Fig. 3b). In the case of *C. elegans*, some pathogenic bacteria that reduce egg hatching also act as repellents [56, 57]. However, *C. elegans* also displays naïve attraction followed by learned avoidance to some pathogenic bacteria, illustrating that pathogenic bacteria do not always elicit a chemorepulsive response [58]. The *S. stercoralis* adults we tested were likely to have previously encountered *P. mirabilis* in host feces and yet were still attracted to it. Thus, whether *S. stercoralis* exhibits learned avoidance to pathogenic bacteria remains unclear.

Our results demonstrate an important role for host-associated and environmental bacteria in the *Strongyloides* life cycle. The robust effects of bacteria on *S. stercoralis* behavior and physiology raise the possibility that bacteria may be an important source of novel nematicidal compounds. In particular, future studies investigating metabolites and other compounds produced by *P. mirabilis* could lead to the identification of natural products with potent nematicidal activity. In addition, understanding the neural and molecular mechanisms that mediate the interactions of parasitic nematodes with bacteria may identify potential targets for disruption and facilitate the development of new strategies for nematode control.

## Conclusions

Our results reveal how *Strongyloides* parasitic nematodes navigate and interact with their microbial environment. We show that the human parasite *Strongyloides stercoralis* is associated with a specific microbiome during its free-living adult life stage. We also show that *Strongyloides* species show dynamic, life-stage-specific behavioral responses to bacteria, suggesting that bacteria represent important chemosensory cues for these parasites. They display robust attraction to many bacteria during their free-living life stage but more specific attraction to certain environmental bacteria during their infective larval stage. Finally, we show that the bacterium *Proteus mirabilis* reduces the parasite’s reproductive capacity, implicating *P. mirabilis* as a possible source of nematicides. Elucidating these microbial interactions informs the development of effective treatment strategies.

## Methods

### *Strongyloides* culture and collection

All protocols and procedures involving vertebrate animals were approved by the UCLA Office of Animal Research Oversight (Protocol 2011-060-31), which adheres to the standards of the AAALAC and the Guide for the Care and Use of Laboratory Animals. *S. stercoralis* strain UPD (originally provided by Dr. James Lok, University of Pennsylvania) was maintained by serial passage in male and female Mongolian gerbils (Charles River Laboratories) as previously described [22, 59]. Briefly, gerbils were anesthetized with isoflurane and inoculated with ~ 2000–2250 iL3s in 200 μL sterile 1x PBS by subcutaneous infection. Between days 14 and 45 post-infection, feces were collected by placing the infected gerbils overnight on wire cage bottoms with wet cardboard underneath. Fecal pellets were mixed with autoclaved charcoal granules (bone char from Ebonex Corp., Cat # EBO.58 BC.04), and stored in Petri dishes lined with dH_2_O-moistened filter paper. *S. ratti* strain ED321 (originally provided by Dr. James Lok, University of Pennsylvania) was maintained by serial passage in female Sprague Dawley rats (Envigo) as previously described [22, 59]. Briefly, rats were inoculated with ~ 800 iL3s in 300 μL sterile 1x PBS by subcutaneous injection. Between days 7 and 23 post-infection, feces were collected as described above. Fecal-charcoal cultures were maintained at 25 °C for 1 day to obtain adults, or at 20 °C for 2 days and then at 23 °C for 6–9 days to obtain iL3s. Worms were then isolated using a Baermann apparatus [18]. For all assays, worms were obtained from at least two sets of host animals and tested across multiple days to account for any day-to-day and batch-to-batch variability.

### *S. stercoralis* DNA extraction for 16S amplicon sequencing

Isolated worms and wash buffer controls were all processed similarly for DNA extraction. *S. stercoralis* young adults or iL3s were isolated from fecal-charcoal plates. For each independent infection experiment (Experiments 1 and 2), gerbil feces were collected close to the peak of infection (days 14–27). In Experiment 1, free-living adults and iL3s were collected from the pooled feces of three different sets of gerbils; worms were collected from the feces of each set of gerbils only once. In Experiment 2, free-living adults were collected from the pooled feces of four different sets of gerbils; worms were collected from the feces of each set of gerbils twice, on two different days. To obtain free-living adults, fecal-charcoal plates were incubated at 20 °C for 2 days; to obtain iL3s, fecal-charcoal plates were incubated at 23 °C for 7 days. Worms were isolated using a Baermann apparatus [18], and then collected from the Baermann funnel in 45 mL of water.

To isolate worms for DNA extraction, 4.5 mL of 100 mM levamisole was added to the 45 mL worm suspension to paralyze the worms. An aliquot containing > 200 worms (Experiment 1) or ~ 25-50 worms (Experiment 2) was removed and centrifuged at 1931 rpm for 2 min to pellet the worms, and all but 100 μL of supernatant was discarded. The worms were then washed 3x in wash buffer consisting of 4 mL of 100 mM levamisole, 4 μL of Triton X-100, and 35.9 mL BU saline [60]. A separate 50 μL aliquot of wash buffer was set aside as a sequencing control (“wash buffer control 1”). After the wash steps, a 50 μL aliquot of supernatant was also set aside as a sequencing control (“wash buffer control 2”). Fifty microliters of worm pellet was transferred to a new tube (“nematode sample”). Two hundred microliters of wash buffer was then added to all samples. Samples were incubated at room temperature for 10 min, and then washed again 3x in wash buffer. After the third wash, samples were spun down and all but 100 μL of sample was discarded. Samples were subjected to bead-beating by vortexing for 5 min with 10–15 sterile garnet particles. The particles were then removed, and DNA was extracted using either the QIAamp DNA Stool Kit (Qiagen; Experiment 1) or a DNA extraction protocol developed for *C. elegans* (Experiment 2) [61].

### Amplicon library construction, sequencing, and data analysis

Barcoded amplicon preparation and sequencing was then performed as previously described [62, 63]. Briefly, supernatants from lysates were transferred to a clean 96-well PCR plate and used as DNA templates. We amplified the V4 region of the 16S rRNA gene using the 515f (GTGYCAGCMGCCGCGGTAA) and 806r (GGACTACNVGGGTWTCTAAT) PCR primers with barcodes added to the 806r primer. Amplicons for each library were normalized based on the PCR product quantified by the image processing package in Fiji [64, 65], then pooled into a single tube. Amplicon products were quantified (Qubit) and then sequenced by the Center for Metagenomics and Microbiome Research, Houston, TX, USA, on an Illumina MiSeq System.

Sequencing datasets were prepared for subsequent statistical analysis using the QIIME2 platform [66]. Samples with fewer than 500 reads were discarded from the analysis. The data was processed with the QIIME2 workflow using the build-in demultiplexing algorithm and Deblur to identify the different Amplicon Sequence Variants (ASVs) present in the datasets. Further statistical analysis was then performed in R using the tidyverse [67], vegan [68], and ANCOM [69] packages. ASVs with a W score in the top 40% of all tested features were selected as significant. The detailed scripts are available at https://bitbucket.org/the-samuel-lab/chavez-2021. Raw reads from the amplicon datasets are available on NCBI at PRJNA678007 (https://identifiers.org/bioproject:PRJNA678007). The following raw data is provided in Additional file 15: Dataset S1: the BIOM file generated by QIIME2 summarizing the ASV distribution per sample for Experiment 1, the BIOM file generated by QIIME2 summarizing the ASV distribution per sample for Experiment 2, the metadata for all sequenced samples, the ASV taxonomies predicted by the QIIME2 pipeline, and the taxonomy summaries at the family level that are depicted in Additional file 1: Fig. S1.

### Bacterial strains, culturing, and maintenance

The bacterial species used were *Proteus mirabilis* (ATCC 29906), *Escherichia fergusonii* (ATCC 35469), *Escherichia coli* HB101 (*Caenorhabditis* Genetics Center), *Pseudomonas fluorescens* (ATCC 13525), *Micrococcus luteus* (ATCC 4698), *Staphylococcus hominis* (ATCC 27844)*, Rhizobium leguminosarum* (ATCC 14479), *Raoultella ornithinolytica* JUb54 and *Raoultella* sp. JUb38 (obtained from Samuel lab, Baylor College of Medicine), and *Bacteroides thetaiotaomicron* (ATCC 29148, obtained from Hsiao lab, UCLA). *E. coli, E. fergusonii, P. mirabilis, P. fluorescens,* JUb38, and JUb54 were maintained on Luria broth (LB) plates, grown in LB, and diluted in LB. *S. hominis* and *M. luteus* were maintained on tryptic soy plates, grown in tryptic soy media, and diluted in tryptic soy media. *R. leguminosarum* was maintained on *Rhizobium* X plates, grown in *Rhizobium* X media, and diluted in *Rhizobium* X media. *B. thetaiotaomicron* was cultured anaerobically in brain-heart infusion supplemented media (BHIS) as previously described [70], and diluted in BHIS. Growth temperatures were 37 °C for all strains except for the *Raoultella* strains, *P. fluorescens*, and *R. leguminosarum* (28 °C); and *M. luteus* (30 °C). Each species was diluted to 1 × 10^8^–1 × 10^9^ CFU/mL as measured by a Thermo Scientific™ NanoDrop™ One spectrophotometer before use.

### Bacterial chemotaxis assays

For standard bacterial chemotaxis assays, 50 μL of bacteria culture, diluted as described above, was plated onto 10 cm 2% Nematode Growth Media (NGM) plates [71] in one of two scoring regions (2 cm diameter circles) on either side of the plate. Fifty microliters of the species-specific media was plated in the other scoring region of the plate as a control (Additional file 7: Fig. S6a). For media control assays (Additional file 7: Fig. S6b-e), a single media type was plated onto both scoring regions. For bacterial competition assays (Additional file 8: Fig. S7), different bacteria were plated onto each scoring region. The plates were then incubated at 37 °C for 30 min. For assays with young adults, worms were collected from a Baermann apparatus and washed twice with BU saline [60] and then twice with dH_2_O in a watch glass before being plated onto the NGM plates. ~ 75–150 worms (or ~ 200–250 worms for media control assays) were plated longitudinally down the center of the plate (Additional file 7: Fig. S6a). For assays with iL3s, the iL3s were collected from a Baermann apparatus and then washed by centrifugation in the solutions described above for 1.5 min at 4400 rpm; ~ 300–400 iL3s were then plated. For *S. ratti* iL3 assays as well as *S. stercoralis* young adult assays with *B. thetaiotaomicron* and the media controls, a glass spreader was used to spread the worms down the center of the plate to reduce clumping. In all cases, the chemotaxis assays were started 2 h after the bacteria were plated and lasted for either 1 h (young adult assays) or 30 min (iL3 assays). Assays were performed at room temperature. For each assay plate, a chemotaxis index (CI) was calculated based on the number of worms in each scoring region: (# worms in bacteria - # worms in control) ÷ (# worms in bacteria + # worms in control). To account for directional bias during the assays, pairs of identical assays were always performed simultaneously with the bacteria placed in scoring regions on opposite sides of the plate. All assays where the absolute difference in CI for the two plates was ≥0.9 were discarded. Assay pairs were also discarded if < 7 total worms moved into the scoring regions on one or both of the paired plates.

### Single-worm tracking assay

Fifty microliters of bacteria culture was plated on a 10 cm 2% NGM plate in 1 of 2 zones (2 cm diameter circles) on either side of the tracking arena, with 3 cm between zones. 50 μL of LB was plated in the other zone as a control. The tracking arena was the 5 cm diameter circle in the center of the 10 cm plate (Additional file 10: Fig. S9). The plates were then incubated at 37 °C for 30 min. After collection from a Baermann apparatus, a small number of *S. stercoralis* free-living adults were removed and placed in a small watch glass with BU. From there a single older, gravid adult female was selected and transferred to dH_2_O. The worm was swirled to wash and then pipetted onto the center of the tracking arena plate in 1–2 μL of dH_2_O. Single-worm tracking was then performed as previously described [23]. The tracking plate was placed above two light diffusers arranged orthogonally on a raised plexiglass surface. The plate surface was bottom-illuminated with a white LED covered with a red-light filter. Tracking was performed in an opaque enclosure to reduce ambient light. Image recording began when the worm left the water droplet. Worm movements were captured using a 5 mega-pixel CMOS camera (BTE-B050-U, Mightex Systems) suspended above the assay plate. Images were saved using the Mightex Cam Demo software (v1.2.1) placed in trigger mode. TTL triggers were provided to the camera at a rate of 0.5 frames/second by a USB DAQ device (U3-LV, LabJack Corp) that was controlled by custom MATLAB (MathWorks) code [59] that was run using MATLAB vR2015b. Images were collected for 20 min or until the worm left the tracking arena.

For post hoc analysis, images were processed into image stacks using a custom FIJI [64] script [59], and worm coordinates on individual images were measured using the Manual Tracking and Cell Counter plugins. The centroids of the experimental (bacteria) and control (LB) zones were measured using FIJI’s Measure command. Worm and zone coordinates (in pixels) and a pixels-to-cm conversion factor were used as inputs to custom MATLAB analysis code [23], selecting the Bacterial Assay Type, that was run using MATLAB v2019b. For individual worms, trajectories were automatically rotated and resized so that the centroids of the experimental and control zones were aligned along the *X*-axis, with an inter-patch distance of 3 cm. To account for directional bias, the location of the bacteria was alternated between left and right zones for each assay. For presentation purposes, tracks in which the bacterial stimulus was placed in the right zone were flipped horizontally to show the bacterial stimulus in the same location in all assays. For LB vs. LB control assays, one zone was randomly assigned as the experimental zone and the other as the control zone. For each experimental condition, individual worm trajectories within the 5 cm diameter assay region were overlaid onto a single plot, along with circles representing the locations of 2 cm diameter zones surrounding the median centroid coordinates of the bacterial (red) and LB (black) stimuli. In addition, the following values were calculated: time spent within the experimental and control zones, median speed, and distance ratio (total track length ÷ maximum displacement). All scripts are available at https://github.com/HallemLab/Chavez_et_al_2021.

### Life span, brood size, and egg hatching assays

To determine survival, brood size, and rates of egg hatching for *S. stercoralis* free-living young adults cultivated on fecal bacteria (Additional file 12: Fig. S11a), assays were performed on 6 cm 2% NGM plates freshly seeded with 50 μL of bacteria, diluted as described above and allowed to dry for at least 30 min. Worms were collected from a Baermann apparatus and washed 3x with BU saline. The worms were then transferred to an unseeded NGM plate and allowed to crawl on the plate for 45 min to remove adhering bacteria. One young adult female containing 0-5 eggs and 3 males were then placed on the bacteria plates. Every 24 h at room temperature, whether the female was alive was determined by examining movement or responsiveness to a gentle prod with a worm pick. The number of eggs and hatched larvae on the plate were also counted. Hatched larvae were defined as those that had at least partially exited the egg case. Living females were then transferred to fresh bacteria plates. Eggs and larvae were counted on days 1–4; progeny counts were only scored for females that were still alive on day 4. Survival was scored on days 1–7, since nearly all worms were dead by day 7.

To compare the rates of egg hatching after 48 h in young versus older adults, bacterial plates were prepared as described above. For assays with young adult females (Additional file 12: Fig. S11b), 1 female containing 0–5 eggs and 3 males were placed onto each bacterial plate. After 24 h at room temperature, the adult worms were removed from the plate, and the number of eggs and larvae were counted to determine the total progeny on the plate. The number of eggs and larvae were counted again at 48 h, and the percentage of hatched eggs was calculated as [(# larvae at 48 h)/(# eggs + # larvae at 24 h)] x 100. For assays with older adults (Additional file 12: Fig. S11c), 4 older, gravid females were placed onto each bacterial plate. The females were then removed after 1.5 h or after at least 20 eggs were laid. The number of eggs was then counted (day 0). After 48 h, the number of larvae on the plate was counted (day 2). The percentage of hatched eggs was then calculated as [(# larvae on day 2)/(# eggs on day 0)] x 100.

To determine whether a soluble factor from *P. mirabilis* inhibits *S. stercoralis* egg hatching, *P. mirabilis* and *E. coli* were grown and diluted as described above. 500 μL of bacterial culture was then spun at 14,800 rpm for 1 min to pellet the bacterial cells, and the supernatant was removed. The supernatants were filter-sterilized using a 0.22 μm syringe-top filter, and 100 μL of *E. coli* or *P. mirabilis* supernatant was then added to 100 μL of *E. coli* liquid culture. Fifty microliters of the bacterial culture was then plated as described above. A young adult 48 h hatching assay was then conducted as described above. To determine whether a volatile factor from *P. mirabilis* inhibits *S. stercoralis* egg hatching, bacterial plates were prepared as described above. A second plate seeded with either *E. coli* or *P. mirabilis* was inverted and placed above the plate with the worms, secured with Parafilm. A young adult 48 h hatching assay was then conducted as described above.

To assess the effects of *P. mirabilis* on egg development, young adult 48 h hatching assays were carried out as outlined above (Additional file 12: Fig. S11b). Plates were scored at 24 h and 48 h, and all plate progeny were categorized according to apparent morphological development. Eggs without distinct morphology (anything prior to the “bean” stage) were classified as “undeveloped,” whereas eggs that developed past this stage were categorized as “developing.” The “developing” group was subcategorized into “bean,” “comma,” “tadpole,” “plum,” and “pretzel” stages; any eggs past the “plum” stage were categorized as “pretzel” (Fig. 6a). Hatched larvae were categorized as healthy or unhealthy. At least 5 eggs needed to be laid by 24 h for the assay to be counted. The images shown in Fig. 6a were taken on a Leica M165 FC fluorescent microscope with an attached Nikon D600 camera.

To determine whether *P. mirabilis* prevents egg hatching indirectly by interfering with mating behavior (Additional file 14: Fig. S13a-b), bacterial plates were prepared as described above. A single young adult female containing 1-5 eggs was placed onto the bacterial plate. A young adult 48 h hatching assay and progeny categorization were conducted as described above and the percentage of eggs that hatched at 24 h and 48 h was quantified. For related 12 h egg lay assays (Additional file 14: Fig. S13c), a similar procedure was used, except that the lone adult female was removed from the plate after 12 h and the percentage of eggs that hatched was calculated at 24 h as [(# larvae at 24 h)/(# eggs + # larvae at 12 h)] × 100.

### Data analysis

Statistical analysis was performed using GraphPad Prism v8.4.2. Violin plots were generated using medium smoothing. Custom MATLAB code was used to analyze and generate single-worm tracks, as described above. For all data sets, the D’Agostino-Pearson omnibus normality test was used to determine if values came from a Gaussian distribution. For data sets that were normally distributed, parametric tests were used. For non-normal distributions, non-parametric tests were used. Statistical tests used and significance thresholds are indicated in figure legends.

## Supplementary Information

**Additional file 1: Fig. S1.** Microbial community profiles for *S. stercoralis* and controls. Stacked bar plots showing the bacterial families that were identified by 16S amplicon sequencing. Sequencing samples included DNA isolated from wash buffer control 1, consisting of the buffer that was used to wash the nematodes; wash buffer control 2, consisting of the buffer supernatant after washing the nematodes; *S. stercoralis* free-living adults; and *S. stercoralis* iL3s (Fig. 2a). Non-significant amplicon sequence variants (ASVs) with < 0.2% abundance across samples were collapsed into the “Other” group. Striped colors indicate genera that are significantly different between categories (ANCOM W > 60% of tested features). Those ASVs are further identified with an asterisk in the legend. The Campylobacteria are a reclassification of the Epsilonproteobacteria and a sister phyla of the Proteobacteria [72].

**Additional file 2: Fig. S2.** Heatmap summary of the genera abundance across samples for Experiment 1. Average relative abundance of different genera found in the different sample categories for Experiment 1. Sequencing samples were as described for Additional file 1: Fig. S1, in addition to an empty well negative control that was processed along with the other samples. ASVs were filtered to display only samples with an average relative abundance > 0.5%. Small columns within each category represent replicate samples.

**Additional file 3: Fig. S3.** Heatmap summary of order abundance across samples for Experiment 1. Average relative abundance of different orders found in the different sample categories for Experiment 1, indicating the large abundance of *Escherichia-Shigella, Lactobacillus,* and *Solibacillus* ASVs in *S. stercoralis* free-living adults. Sequencing samples were as described for Additional file 1: Fig. S1, in addition to an empty well negative control that was processed along with the other samples. ASVs were filtered to display only samples with an average relative abundance > 0.5%. ASV identifiers in red are those that showed a significant enrichment in *S. stercoralis* free-living adults vs. controls in both sequencing experiments. Small columns within each category represent replicate samples.

**Additional file 4: Fig. S4.** Heatmap summary of the genera abundance across samples for Experiment 2. Average relative abundance of different genera found in the different sample categories for Experiment 2. Sequencing samples were as described for Additional file 1: Fig. S1, except that *S. stercoralis* iL3s were not included in this experiment. ASVs were filtered to display only samples with an average relative abundance > 0.5%. Small columns within each category represent replicate samples.

**Additional file 5: Fig. S5.** Heatmap summary of order abundance across samples for Experiment 2. Average relative abundance of different orders found in the different sample categories for Experiment 2, indicating the large abundance of *Escherichia-Shigella, Lactobacillus,* and *Solibacillus* ASVs in *S. stercoralis* free-living adults. Sequencing samples were as described for Additional file 1: Fig. S1, except that *S. stercoralis* iL3s were not included in this experiment. ASVs were filtered to display only samples with an average relative abundance > 0.5%. ASV identifiers in red are those that showed a significant enrichment in *S. stercoralis* free-living adults vs. controls in both sequencing experiments. Small columns within each category represent replicate samples.

**Additional file 6: Table S1.** The bacterial panel selected. The table lists each bacterial species tested, its environment-type designation, the rationale for selecting it, and supporting references.

**Additional file 7: Fig. S6.** Bacteria chemotaxis assay design. a. Diagram of the bacterial chemotaxis assay. Following plating of bacteria and unseeded media on either side of the plate, a population of nematodes was placed along the center of a 10 cm 2% NGM plate and allowed to migrate for the duration of the assay. A chemotaxis index (CI) was then calculated after counting the number of nematodes in each region as: CI = (# worms in bacteria - # worms in control) / (# worms in bacteria + # worms in control). A positive CI indicates attraction to the bacteria, a negative CI indicates repulsion, and a CI near zero indicates a neutral response. b-e. Control chemotaxis assays with unseeded media on both sides of the plate. Each point in the graphs shows the CI of a single trial; medians (solid lines) and interquartile ranges (dashed lines) are also shown. LB = Luria broth; TS = tryptic soy media; RhiX = *Rhizobium* X media; BHIS = brain-heart infusion supplemented media. For each graph, no significant differences were detected comparing each condition to all other conditions, Kruskal-Wallis test with Dunn’s post-test (b and e) or Brown-Forsythe and Welch ANOVA with Dunnett’s T3 post-test (c-d). *n* = 20-32 trials per condition.

**Additional file 8: Fig. S7.** *S. stercoralis* free-living adults do not display strong preferences among fecal/gut bacteria. a. Diagram of the bacterial chemotaxis assay. For bacterial competition chemotaxis assays, different bacteria were plated on each side of the plate. A chemotaxis index (CI) was calculated after counting the number of nematodes in each region as: CI = (# worms in bacteria 1 region - # worms in bacteria 2 region) / (# worms in bacteria 1 region + # worms in bacteria 2 region). b. *S. stercoralis* free-living adults did not prefer *E. coli* or *E. fergusonii* over *P. mirabilis*. No significant differences were detected (Brown-Forsythe and Welch ANOVA). c. *S. stercoralis* free-living adults showed a slight preference for *E. coli* over *E. fergusonii*. **p< 0.01, Brown-Forsythe and Welch ANOVA with Dunnett’s T3 post-test. Only the significant difference is noted. For b-c, each condition was compared to the control condition where the same bacterial species was plated on both sides of the plate. The same data are represented in b and c. *n* = 24-38 trials per condition, with 75-150 worms per trial. Each point in the graphs shows the chemotaxis index of a single trial; medians (solid lines) and interquartile ranges (dashed lines) are also shown. For each condition, the bacteria listed at the top and bottom of the graph indicate the two bacteria being tested in the competition assay.

**Additional file 9.: Fig. S8.** *S. ratti* and *S. stercoralis* respond similarly to the bacterial species tested. a. *S. ratti* free-living adults and *S. stercoralis* free-living adults displayed similar chemotaxis responses to the bacterial panel. No significant differences were detected (two-way ANOVA with Sidak’s post-test). *n* = 20-40 trials per condition, with 75-150 worms per trial. b. *S. ratti* iL3s and *S. stercoralis* iL3s displayed similar chemotaxis responses to the bacterial panel, with only a minor difference in the response to *P. mirabilis*. *p< 0.05, two-way ANOVA with Sidak’s post-test. Only the significant difference is noted. *n* = 20-30 trials per condition, with 300-400 worms per trial. Each point in the graphs shows the chemotaxis index of a single trial; medians (solid lines) and interquartile ranges (dashed lines) are also shown. Data are from Fig. 3.

**Additional file 10: Fig. S9.** Single-worm tracking assay design. Bacteria and unseeded LB media were plated on either side of a 5 cm diameter tracking arena (bolded inner circle) centered on a 10 cm 2% NGM plate (left). A single, older *S. stercoralis* free-living adult female was placed in the center of the arena and recorded for 20 min or until it left the tracking arena. Video analysis software and custom MATLAB code (see Materials and Methods) were used to produce and compile movement tracks (right). Pink and grey shaded circles indicate the location of the bacteria and LB media, respectively; red and grey circle outlines indicate the experimental region and the control region, respectively.

**Additional file 11: Fig. S10.** *S. stercoralis* free-living adults move through different bacteria at similar speeds. Median crawling speeds only through the bacterial lawns were analyzed from single-worm tracking data. No significant differences were detected comparing each condition to every other condition (Kruskal-Wallis test with Dunn’s post-test). *n* = 5-16 worms per condition. Graph shows medians (solid lines) and interquartile ranges (dashed lines).

**Additional file 12: Fig. S11.** Life span, brood size, and egg hatching assay design. a. To measure life span, brood size, and egg hatching after 24 hours, three free-living adult *S. stercoralis* males and one free-living young adult female were placed on a plate containing either *E. coli*, *E. fergusonii*, or *P. mirabilis* (day 0). On days 1-7, survival of the female was scored every 24 h, and if the female was still alive, it was transferred to a fresh bacterial plate. On days 1-4, after transferring the female to a fresh plate, the numbers of eggs and larvae present on the plate the female was transferred off of were recorded. On days 5-7, only survival was monitored. Brood size and egg hatching were calculated only for females that were still alive on day 4. b. To measure the rate of egg hatching after 48 h with young adult females, three free-living adult *S. stercoralis* males and one free-living young adult female were placed on a bacteria plate. All adults were removed after 24 h. The number of eggs and larvae present on the plate were recorded at 24 h and 48 h to determine the percentage of eggs that had hatched after 48 h. c. To measure the rate of egg hatching after 48 h with older females, four older free-living adult *S. stercoralis* females were placed on bacteria plates. All adults were removed after 1.5 h or after 20 eggs were laid (0 h time point). Eggs were recorded at 0 h and larvae at 48 h to determine the rate of egg hatching after 48 h.

**Additional file 13: Fig. S12.** Neither soluble nor volatile *P. mirabilis* factors are sufficient to decrease *S. stercoralis* egg hatching. a. No significant differences in the rates of egg hatching after 48 h were observed when *S. stercoralis* young adult females were cultured on either *E. coli* supplemented with *E. coli* supernatant or *E. coli* supplemented with *P. mirabilis* supernatant (unpaired two-tailed Welch’s t test). *n* = 9 trials per condition. Graph shows medians (solid lines) and interquartile ranges (dashed lines). b. No significant differences in the rates of egg hatching after 48 h were observed when *S. stercoralis* young adult females were cultured on *E. coli* either in the presence of *E. coli* volatiles or in the presence of *P. mirabilis* volatiles (two-tailed Mann-Whitney test). *n* = 5-6 trials per condition. Graph shows medians (solid lines) and interquartile ranges (dashed lines). Each assay consisted of 3 *S. stercoralis* males and 1 young adult female; adults were removed after 24 h, and the percentage of eggs that had hatched was determined at 48 h.

**Additional file 14: Fig. S13.** The effect of *P. mirabilis* on *S. stercoralis* egg hatching is due to a direct effect on embryonic development rather than an indirect effect on mating behavior. a. Culturing individual *S. stercoralis* young adult females on *P. mirabilis* in the absence of males resulted in reduced egg hatching after 24 h (left) and 48 h (right). For these experiments, lone young adult females containing 1-5 eggs in their gonad were cultured on a lawn of either *P. mirabilis* or *E. coli* in the absence of males for 24 h, and the frequency of egg hatching was then scored at 24 and 48 h. ***p< 0.001, *p< 0.05, Mann Whitney test (left) or Welch’s t-test (right). *n* = 8 trials per condition. b. Culturing individual *S. stercoralis* young adult females on *P. mirabilis* in the absence of males resulted in decreased frequencies of healthy larvae. For these experiments, lone young adult females containing 1-5 eggs in their gonad were cultured as described above, and eggs and larvae were scored at 24 and 48 h as described in Fig. 6. *p< 0.05, **p< 0.01, ****p< 0.0001, two-way ANOVA with Tukey’s multiple comparisons test. Comparisons are between the same category and time point; only significant differences are shown. *n* = 8-7 trials per condition. Median percentages are represented, and bars represent interquartile ranges. Data in a and b are from the same experiments. c. Culturing individual *S. stercoralis* young adult females on *P. mirabilis* for only 12 h in the absence of males did not result in a decrease in egg hatching, suggesting that *P. mirabilis* impedes egg development specifically in later-laid eggs by interfering with early egg development. ns = not significant (*p*=0.2687), Welch’s t test. *n* = 6-20 trials per condition.

**Additional file 15: Dataset S1.** Microbiome Sequencing Dataset. This dataset contains the BIOM file generated by QIIME2 summarizing the ASV distribution per sample for Experiment 1 (tab 1), the BIOM file generated by QIIME2 summarizing the ASV distribution per sample for Experiment 2 (tab 2), the relevant sample metadata (tab 3), the ASV taxonomies predicted by the QIIME2 pipeline (tab 4), and the taxonomy summaries at the family level that are depicted in Additional file 1: Fig. S1 (tab 5).

## Data Availability

All data from this study are included in this published article and its additional files. All code used for data analysis in this study is publicly available through the Hallem lab GitHub site (https://github.com/HallemLab/Chavez_et_al_2021) or the Samuel lab Bitbucket site (https://bitbucket.org/the-samuel-lab/chavez-2021). Raw reads from the amplicon datasets are available on NCBI at PRJNA678007 (https://identifiers.org/bioproject:PRJNA678007).
