# Supplementary figures and images for: Skin-penetrating nematodes exhibit life-stage-specific interactions with host-associated and environmental bacteria
Source: BMC Biol. 2021 Oct 7;19:221. doi: 10.1186/s12915-021-01153-7 (PMC8499433; doi:10.1186/s12915-021-01153-7)

Additional file 1: Fig. S1

Experiment 1 - Taxonomy barplot

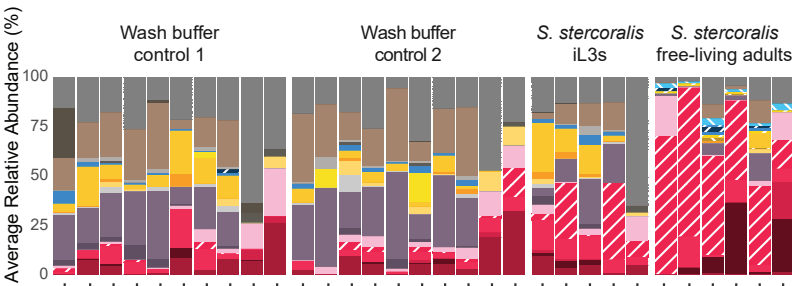

Experiment 2 - Taxonomy barplot

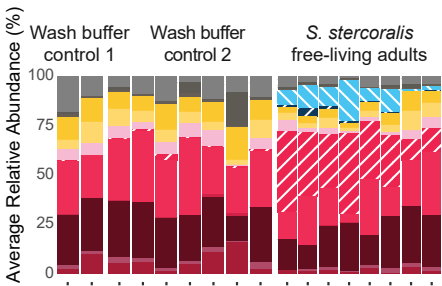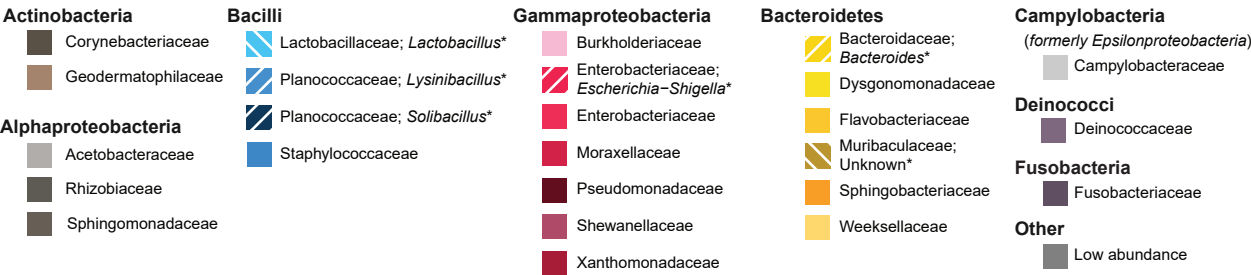

Supplement: Supplementary file 1 — Additional file 1: Fig. S1. Microbial community profiles for S. stercoralis and controls. Stacked bar plots showing the bacterial families that were identified by 16S amplicon sequencing. Sequencing samples included DNA isolated from wash buffer control 1, consisting of the buffer that was used to wash the nematodes; wash buffer control 2, consisting of the buffer supernatant after washing the nematodes; S. stercoralis free-living adults; and S. stercoralis iL3s (Fig. 2a). Non-significant amplicon sequence variants (ASVs) with < 0.2% abundance across samples were collapsed into the “Other” group. Striped colors indicate genera that are significantly different between categories (ANCOM W > 60% of tested features). Those ASVs are further identified with an asterisk in the legend. The Campylobacteria are a reclassification of the Epsilonproteobacteria and a sister phyla of the Proteobacteria [72]. [file 12915_2021_1153_MOESM1_ESM.pdf]

Additional file 2: Fig. S2

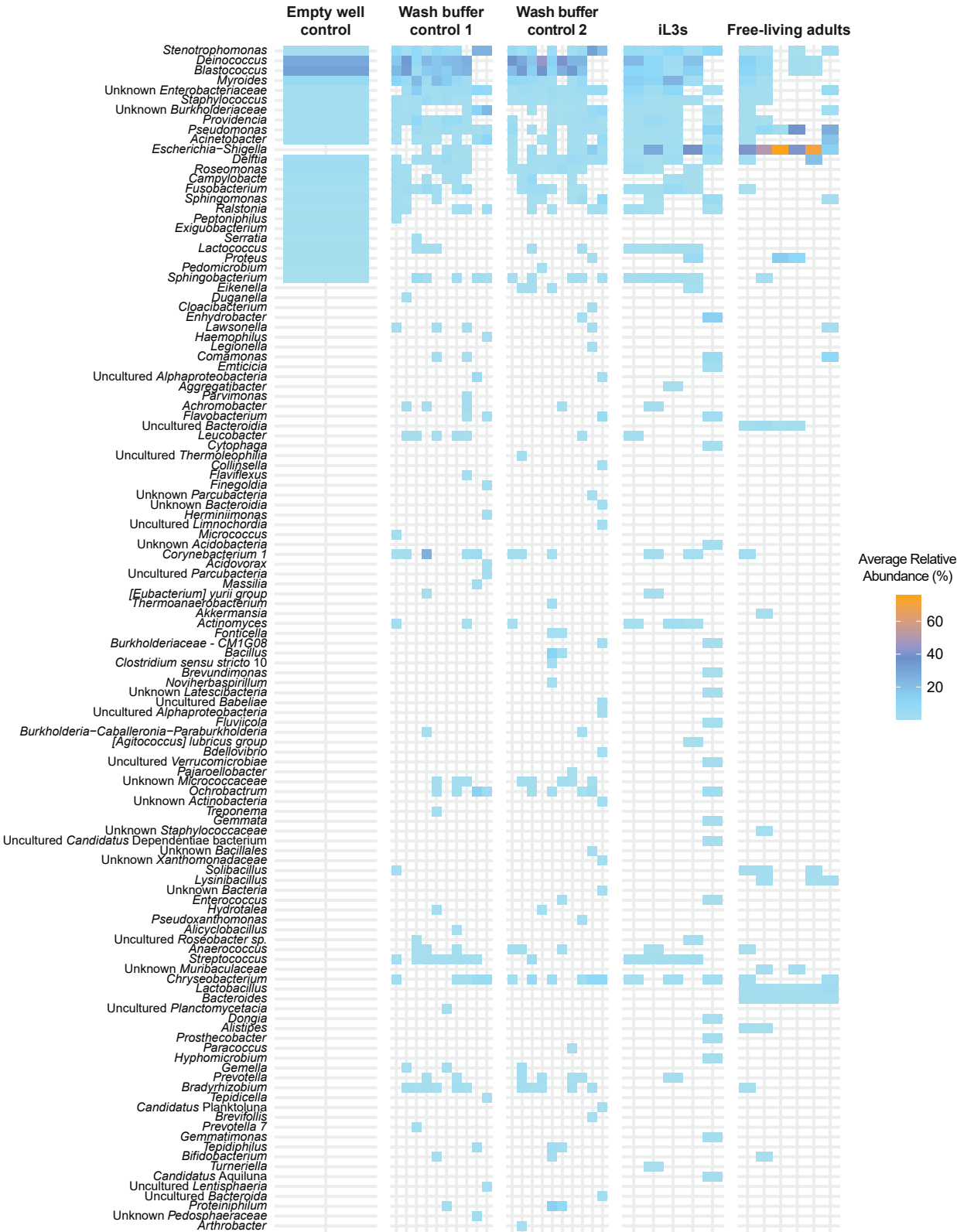

Supplement: Supplementary file 2 — Additional file 2: Fig. S2. Heatmap summary of the genera abundance across samples for Experiment 1. Average relative abundance of different genera found in the different sample categories for Experiment 1. Sequencing samples were as described for Additional file 1: Fig. S1, in addition to an empty well negative control that was processed along with the other samples. ASVs were filtered to display only samples with an average relative abundance > 0.5%. Small columns within each category represent replicate samples. [file 12915_2021_1153_MOESM2_ESM.pdf]

Additional file 3: Fig. S3

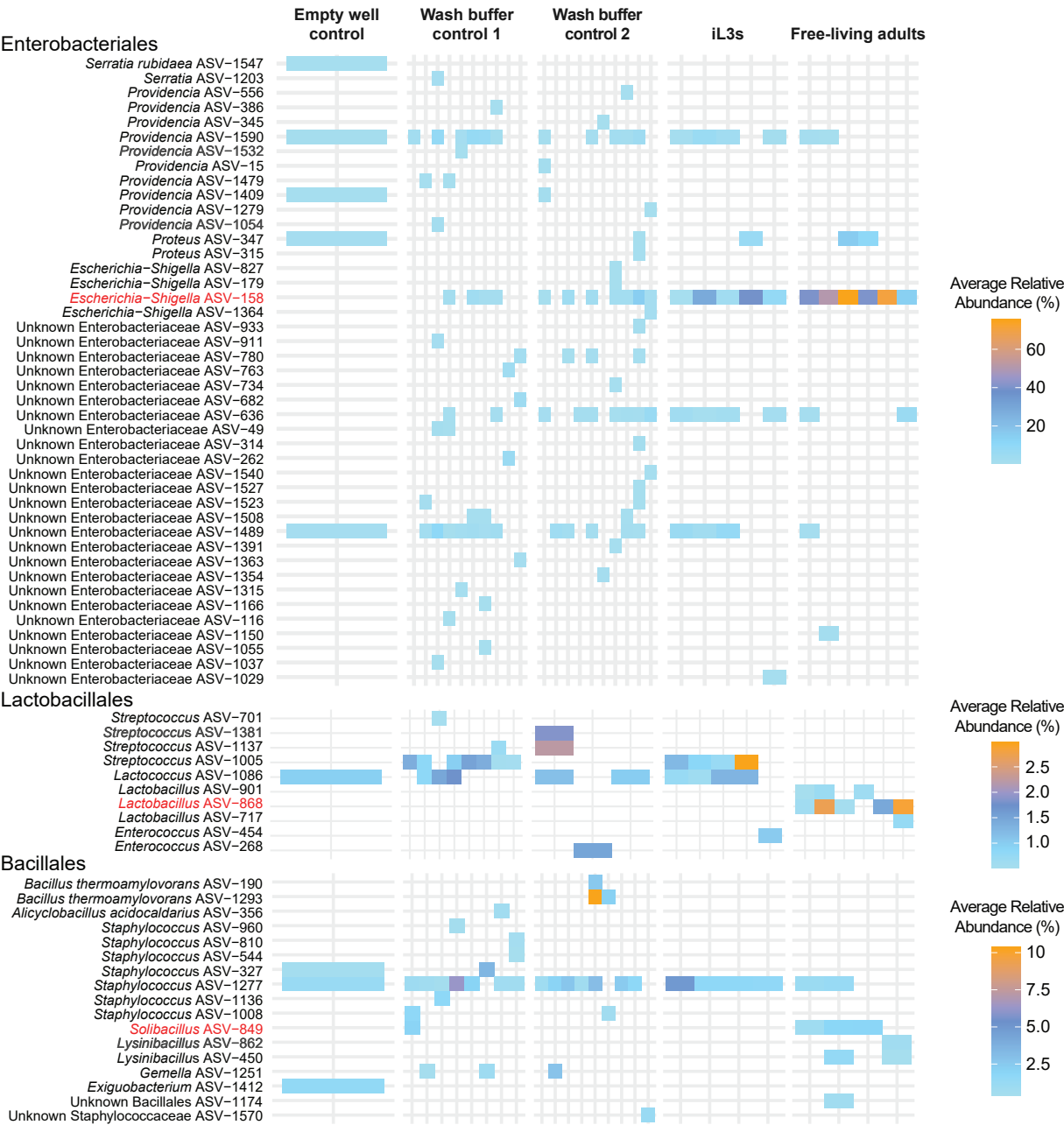

Supplement: Supplementary file 3 — Additional file 3: Fig. S3. Heatmap summary of order abundance across samples for Experiment 1. Average relative abundance of different orders found in the different sample categories for Experiment 1, indicating the large abundance of Escherichia-Shigella, Lactobacillus, and Solibacillus ASVs in S. stercoralis free-living adults. Sequencing samples were as described for Additional file 1: Fig. S1, in addition to an empty well negative control that was processed along with the other samples. ASVs were filtered to display only samples with an average relative abundance > 0.5%. ASV identifiers in red are those that showed a significant enrichment in S. stercoralis free-living adults vs. controls in both sequencing experiments. Small columns within each category represent replicate samples. [file 12915_2021_1153_MOESM3_ESM.pdf]

Additional file 4: Fig. S4

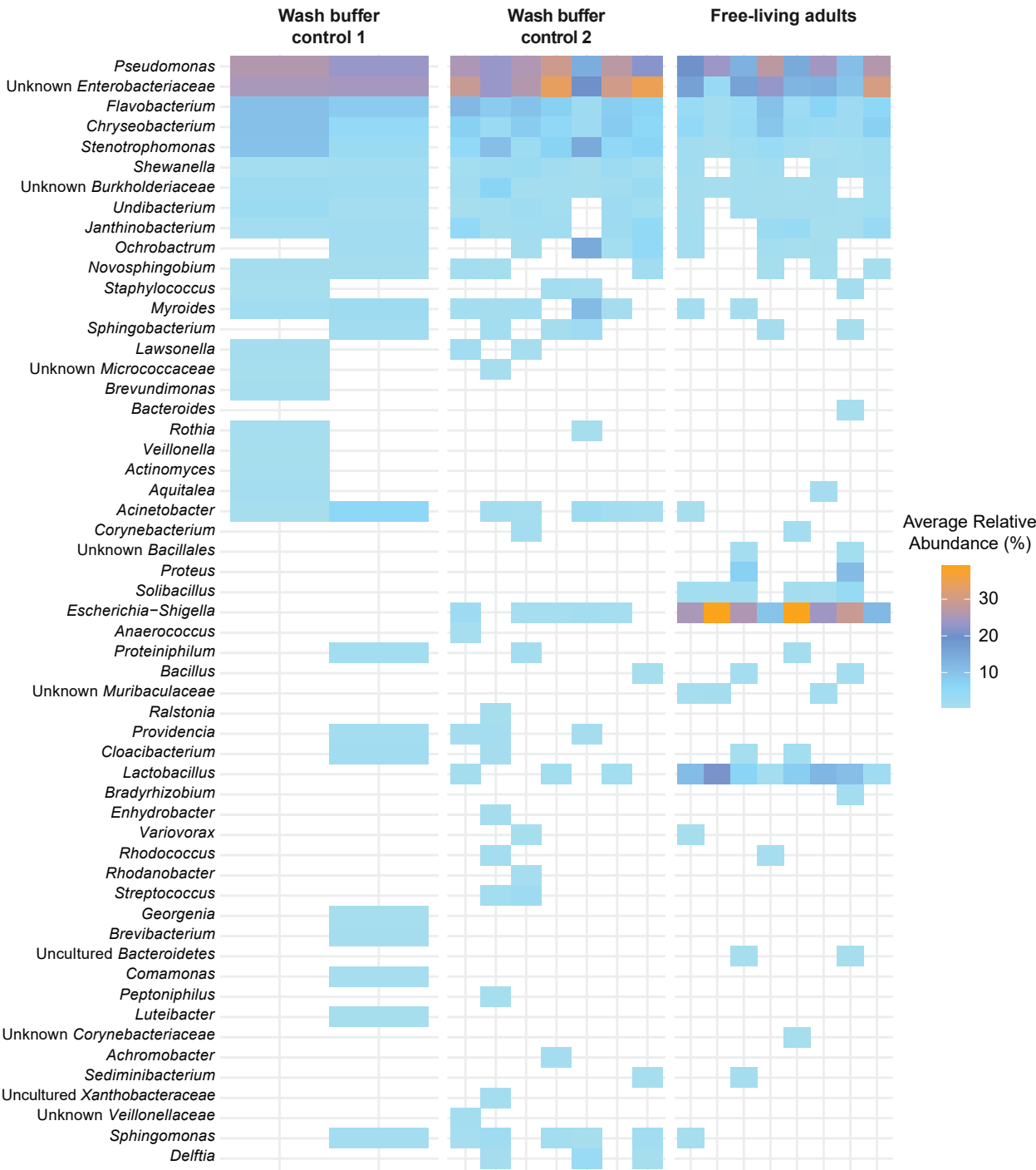

Supplement: Supplementary file 4 — Additional file 4: Fig. S4. Heatmap summary of the genera abundance across samples for Experiment 2. Average relative abundance of different genera found in the different sample categories for Experiment 2. Sequencing samples were as described for Additional file 1: Fig. S1, except that S. stercoralis iL3s were not included in this experiment. ASVs were filtered to display only samples with an average relative abundance > 0.5%. Small columns within each category represent replicate samples. [file 12915_2021_1153_MOESM4_ESM.pdf]

Additional file 5: Fig. S5

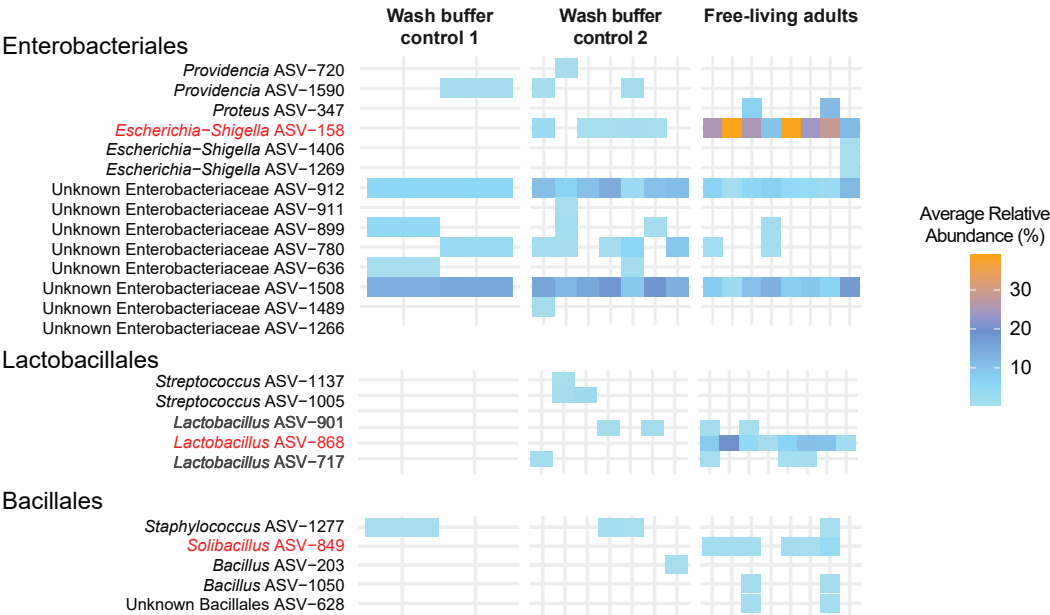

Supplement: Supplementary file 5 — Additional file 5: Fig. S5. Heatmap summary of order abundance across samples for Experiment 2. Average relative abundance of different orders found in the different sample categories for Experiment 2, indicating the large abundance of Escherichia-Shigella, Lactobacillus, and Solibacillus ASVs in S. stercoralis free-living adults. Sequencing samples were as described for Additional file 1: Fig. S1, except that S. stercoralis iL3s were not included in this experiment. ASVs were filtered to display only samples with an average relative abundance > 0.5%. ASV identifiers in red are those that showed a significant enrichment in S. stercoralis free-living adults vs. controls in both sequencing experiments. Small columns within each category represent replicate samples. [file 12915_2021_1153_MOESM5_ESM.pdf]

Additional file 7: Fig. S6

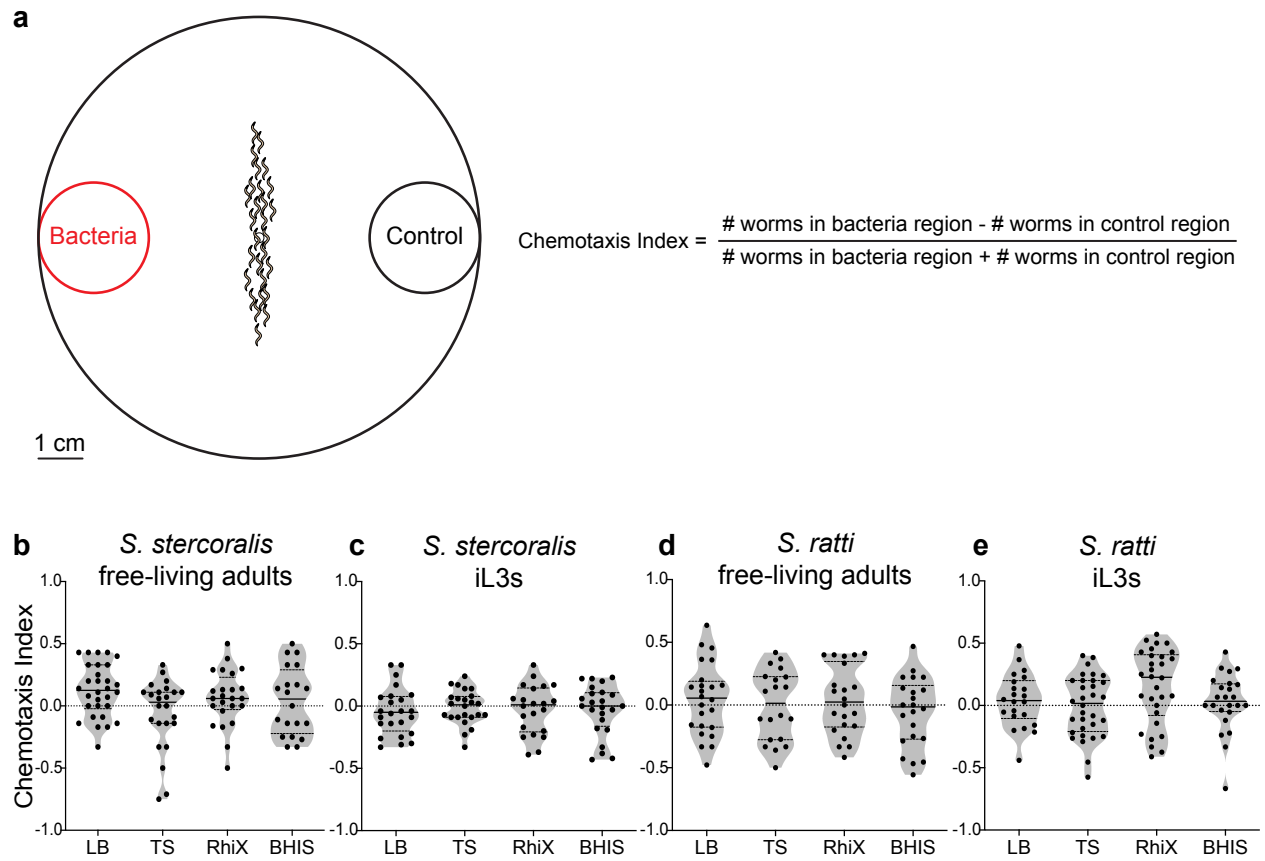

Supplement: Supplementary file 7 — Additional file 7: Fig. S6. Bacteria chemotaxis assay design. a. Diagram of the bacterial chemotaxis assay. Following plating of bacteria and unseeded media on either side of the plate, a population of nematodes was placed along the center of a 10 cm 2% NGM plate and allowed to migrate for the duration of the assay. A chemotaxis index (CI) was then calculated after counting the number of nematodes in each region as: CI = (# worms in bacteria - # worms in control) / (# worms in bacteria + # worms in control). A positive CI indicates attraction to the bacteria, a negative CI indicates repulsion, and a CI near zero indicates a neutral response. b-e. Control chemotaxis assays with unseeded media on both sides of the plate. Each point in the graphs shows the CI of a single trial; medians (solid lines) and interquartile ranges (dashed lines) are also shown. LB = Luria broth; TS = tryptic soy media; RhiX = Rhizobium X media; BHIS = brain-heart infusion supplemented media. For each graph, no significant differences were detected comparing each condition to all other conditions, Kruskal-Wallis test with Dunn’s post-test (b and e) or Brown-Forsythe and Welch ANOVA with Dunnett’s T3 post-test (c-d). n = 20-32 trials per condition. [file 12915_2021_1153_MOESM7_ESM.pdf]

Additional file 9: Fig. S8

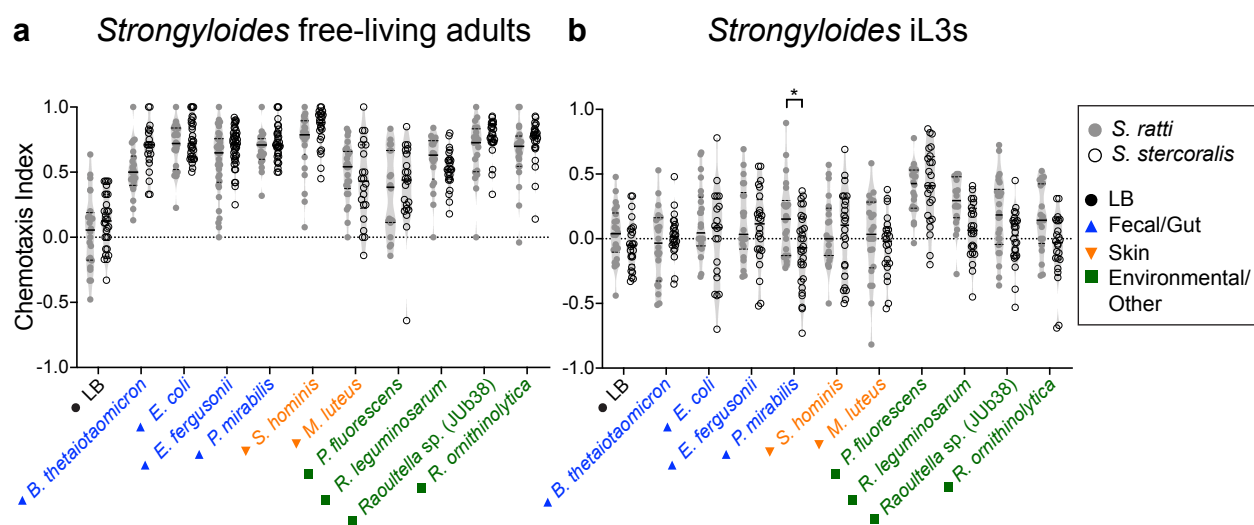

Supplement: Supplementary file 9 — Additional file 9.: Fig. S8. S. ratti and S. stercoralis respond similarly to the bacterial species tested. a. S. ratti free-living adults and S. stercoralis free-living adults displayed similar chemotaxis responses to the bacterial panel. No significant differences were detected (two-way ANOVA with Sidak’s post-test). n = 20-40 trials per condition, with 75-150 worms per trial. b. S. ratti iL3s and S. stercoralis iL3s displayed similar chemotaxis responses to the bacterial panel, with only a minor difference in the response to P. mirabilis. *p< 0.05, two-way ANOVA with Sidak’s post-test. Only the significant difference is noted. n = 20-30 trials per condition, with 300-400 worms per trial. Each point in the graphs shows the chemotaxis index of a single trial; medians (solid lines) and interquartile ranges (dashed lines) are also shown. Data are from Fig. 3. [file 12915_2021_1153_MOESM9_ESM.pdf]

Additional file 10: Fig. S9

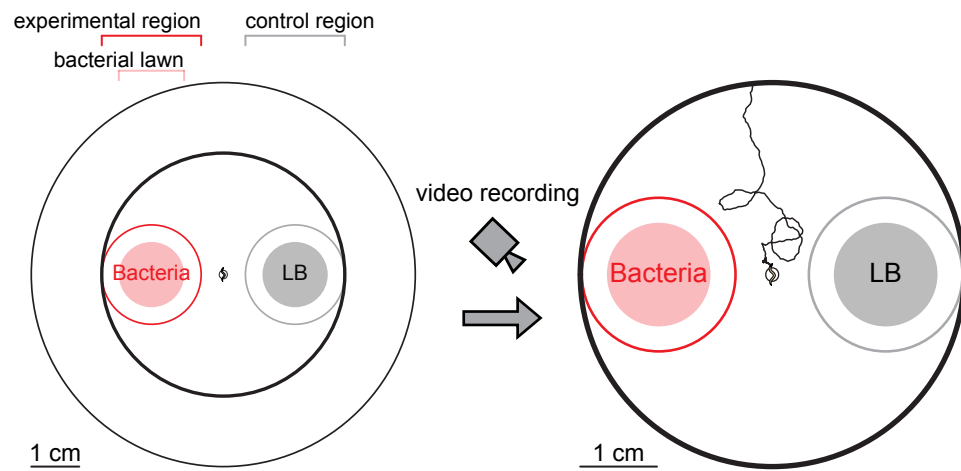

Supplement: Supplementary file 10 — Additional file 10: Fig. S9. Single-worm tracking assay design. Bacteria and unseeded LB media were plated on either side of a 5 cm diameter tracking arena (bolded inner circle) centered on a 10 cm 2% NGM plate (left). A single, older S. stercoralis free-living adult female was placed in the center of the arena and recorded for 20 min or until it left the tracking arena. Video analysis software and custom MATLAB code (see Materials and Methods) were used to produce and compile movement tracks (right). Pink and grey shaded circles indicate the location of the bacteria and LB media, respectively; red and grey circle outlines indicate the experimental region and the control region, respectively. [file 12915_2021_1153_MOESM10_ESM.pdf]

Additional file 11: Fig. S10

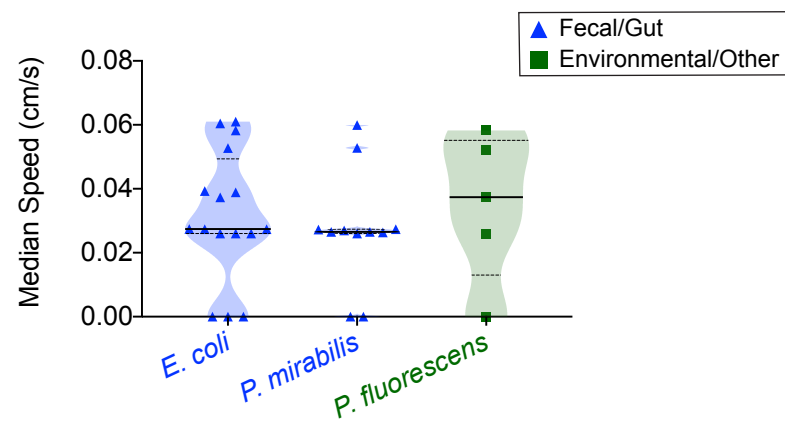

Supplement: Supplementary file 11 — Additional file 11: Fig. S10. S. stercoralis free-living adults move through different bacteria at similar speeds. Median crawling speeds only through the bacterial lawns were analyzed from single-worm tracking data. No significant differences were detected comparing each condition to every other condition (Kruskal-Wallis test with Dunn’s post-test). n = 5-16 worms per condition. Graph shows medians (solid lines) and interquartile ranges (dashed lines). [file 12915_2021_1153_MOESM11_ESM.pdf]

Additional file 12: Fig. S11

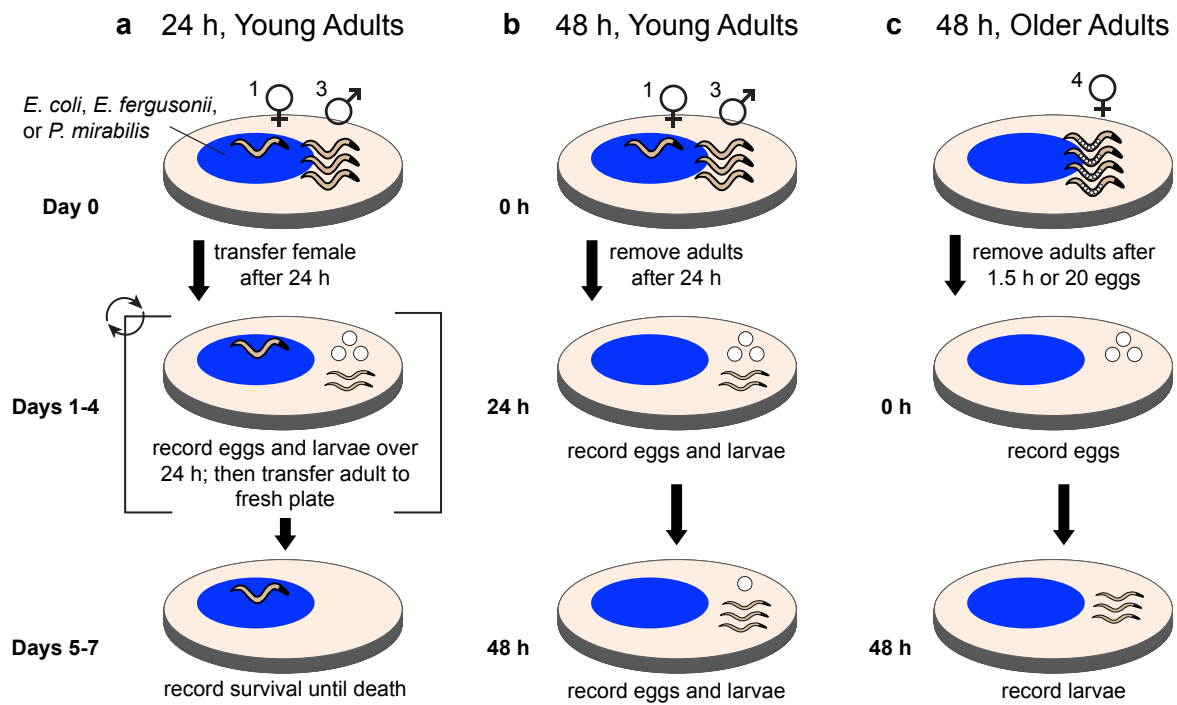

Supplement: Supplementary file 12 — Additional file 12: Fig. S11. Life span, brood size, and egg hatching assay design. a. To measure life span, brood size, and egg hatching after 24 hours, three free-living adult S. stercoralis males and one free-living young adult female were placed on a plate containing either E. coli, E. fergusonii, or P. mirabilis (day 0). On days 1-7, survival of the female was scored every 24 h, and if the female was still alive, it was transferred to a fresh bacterial plate. On days 1-4, after transferring the female to a fresh plate, the numbers of eggs and larvae present on the plate the female was transferred off of were recorded. On days 5-7, only survival was monitored. Brood size and egg hatching were calculated only for females that were still alive on day 4. b. To measure the rate of egg hatching after 48 h with young adult females, three free-living adult S. stercoralis males and one free-living young adult female were placed on a bacteria plate. All adults were removed after 24 h. The number of eggs and larvae present on the plate were recorded at 24 h and 48 h to determine the percentage of eggs that had hatched after 48 h. c. To measure the rate of egg hatching after 48 h with older females, four older free-living adult S. stercoralis females were placed on bacteria plates. All adults were removed after 1.5 h or after 20 eggs were laid (0 h time point). Eggs were recorded at 0 h and larvae at 48 h to determine the rate of egg hatching after 48 h. [file 12915_2021_1153_MOESM12_ESM.pdf]

Additional file 13: Fig. S12

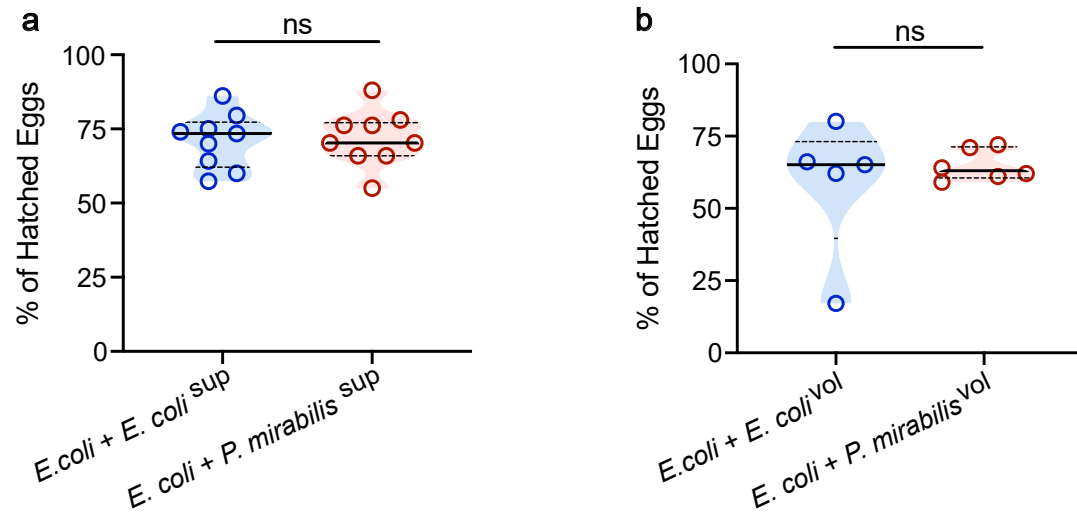

Supplement: Supplementary file 13 — Additional file 13: Fig. S12. Neither soluble nor volatile P. mirabilis factors are sufficient to decrease S. stercoralis egg hatching. a. No significant differences in the rates of egg hatching after 48 h were observed when S. stercoralis young adult females were cultured on either E. coli supplemented with E. coli supernatant or E. coli supplemented with P. mirabilis supernatant (unpaired two-tailed Welch’s t test). n = 9 trials per condition. Graph shows medians (solid lines) and interquartile ranges (dashed lines). b. No significant differences in the rates of egg hatching after 48 h were observed when S. stercoralis young adult females were cultured on E. coli either in the presence of E. coli volatiles or in the presence of P. mirabilis volatiles (two-tailed Mann-Whitney test). n = 5-6 trials per condition. Graph shows medians (solid lines) and interquartile ranges (dashed lines). Each assay consisted of 3 S. stercoralis males and 1 young adult female; adults were removed after 24 h, and the percentage of eggs that had hatched was determined at 48 h. [file 12915_2021_1153_MOESM13_ESM.pdf]

Additional File 14: Fig. S13

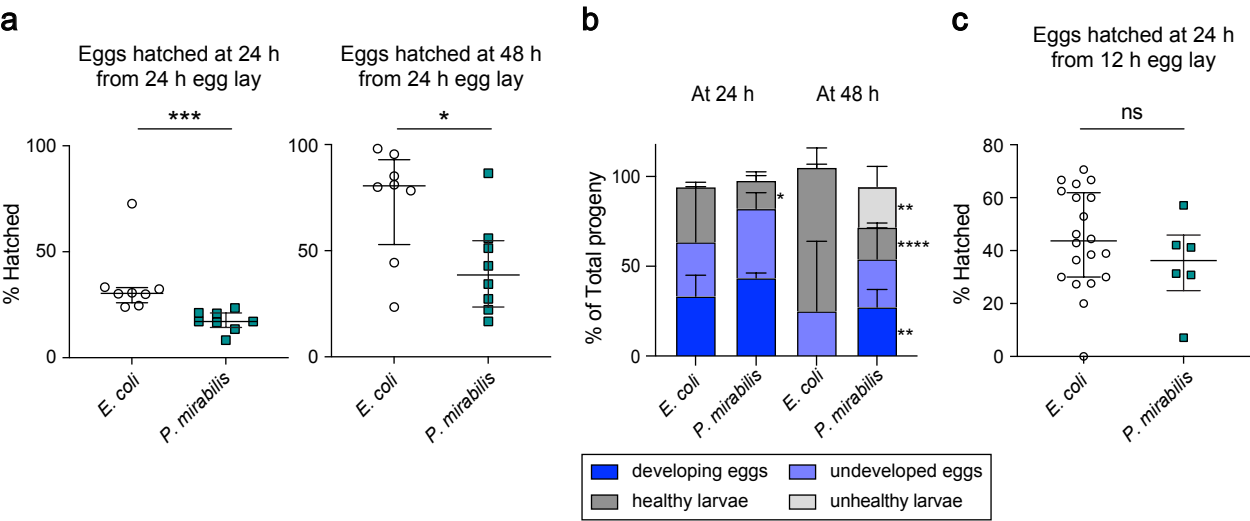

Supplement: Supplementary file 14 — Additional file 14: Fig. S13. The effect of P. mirabilis on S. stercoralis egg hatching is due to a direct effect on embryonic development rather than an indirect effect on mating behavior. a. Culturing individual S. stercoralis young adult females on P. mirabilis in the absence of males resulted in reduced egg hatching after 24 h (left) and 48 h (right). For these experiments, lone young adult females containing 1-5 eggs in their gonad were cultured on a lawn of either P. mirabilis or E. coli in the absence of males for 24 h, and the frequency of egg hatching was then scored at 24 and 48 h. ***p< 0.001, *p< 0.05, Mann Whitney test (left) or Welch’s t-test (right). n = 8 trials per condition. b. Culturing individual S. stercoralis young adult females on P. mirabilis in the absence of males resulted in decreased frequencies of healthy larvae. For these experiments, lone young adult females containing 1-5 eggs in their gonad were cultured as described above, and eggs and larvae were scored at 24 and 48 h as described in Fig. 6. *p< 0.05, **p< 0.01, ****p< 0.0001, two-way ANOVA with Tukey’s multiple comparisons test. Comparisons are between the same category and time point; only significant differences are shown. n = 8-7 trials per condition. Median percentages are represented, and bars represent interquartile ranges. Data in a and b are from the same experiments. c. Culturing individual S. stercoralis young adult females on P. mirabilis for only 12 h in the absence of males did not result in a decrease in egg hatching, suggesting that P. mirabilis impedes egg development specifically in later-laid eggs by interfering with early egg development. ns = not significant (p=0.2687), Welch’s t test. n = 6-20 trials per condition. [file 12915_2021_1153_MOESM14_ESM.pdf]
