# Supplementary material for: Skin-penetrating nematodes exhibit life-stage-specific interactions with host-associated and environmental bacteria
Source: BMC Biol. 2021 Oct 7;19:221. doi: 10.1186/s12915-021-01153-7 (PMC8499433; doi:10.1186/s12915-021-01153-7)
Supplement: Supplementary file 6 — Additional file 6: Table S1. The bacterial panel selected. The table lists each bacterial species tested, its environment-type designation, the rationale for selecting it, and supporting references. [file 12915_2021_1153_MOESM6_ESM.pdf]

Additional file 6: Table S1. The bacterial panel selected. The table lists each bacterial species tested, its environment-type designation, the rationale for selecting it, and supporting references.

**Bacteria**	**Category**	**Selection Rationale**	**References**
Bacteroides thetaiotaomicron (ATCC 29148)	Fecal / Gut	Highly abundant, aerotolerant gut bacterium. *Bacteroides* species found to be associated with *S. stercoralis* free-living adults (Fig. 2).	[1]
Escherichia coli (HB101)	Fecal / Gut	Standard lab nutrient source for nematode propagation. Found in mammalian feces. Associated with sepsis in strongyloidiasis patients. *Escherichia* species found to be associated with *S. stercoralis* free-living adults (Fig. 2).	[2-4]
Escherichia fergusonii (ATCC 35469)	Fecal / Gut	Found in feces. Closely related to *E. coli.* Isolated from human wound infections and mammalian feces. *Escherichia* species found to be associated with *S. stercoralis* free-living adults (Fig. 2).	[5]
Proteus mirabilis (ATCC 29906)	Fecal / Gut	Highly abundant in human feces. Also found in many other places, including soil, stagnant water, and the human intestine. Associated with sepsis in strongyloidiasis patients. Bacteria of the same class (Gammaproteobacteria) found to be associated with *S. stercoralis* free-living adults (Fig. 2).	[2, 6]
Pseudomonas fluorescens (ATCC 13525)	Environmental / Other	Bacterium commonly associated with environmental soil, plants, and water. Found in tropical soils. Also found more broadly in the environment. Bacteria of the same class (Gammaproteobacteria) found to be associated with *S. stercoralis* free-living adults (Fig. 2).	[7, 8]
Rhizobium leguminosarum (ATCC 14479)	Environmental / Other	Bacterium commonly found in soil rhizosphere associated with legumes. Also found more broadly in the environment.	[9]
Micrococcus luteus (ATCC 4698)	Skin	Component of the human skin microbiome.	[10, 11]

Staphylococcus hominis<br><br>(ATCC 27844)	Skin	Component of the human skin microbiome.	[10, 12]
Raoultella sp. (JUb38)	Environmental / Other	Isolated from rotting apple.	[13]
Raoultella ornithinolytica<br><br>(JUb54)	Environmental / Other	Isolated from rotting apple.	[13]

References:

1. Comstock LE, Coyne MJ: ***Bacteroides thetaiotaomicron*: a dynamic, niche-adapted human symbiont.** *Bioessays* 2003, **25**(10):926-929.

2. Nutman TB: **Human infection with *Strongyloides stercoralis* and other related *Strongyloides* species**. *Parasitology* 2017, **144**(3):263-273.

3. Lok JB: ***Strongyloides stercoralis:* a model for translational research on parasitic nematode biology**. In *WormBook* 2007, www.wormbook.org, p. 1-18.

4. Ghoshal UC, Ghoshal U, Jain M, Kumar A, Aggarwal R, Misra A, Ayyagari A: ***Strongyloides stercoralis* infestation associated with septicemia due to intestinal transmural migration of bacteria**. *J Gastroenterol Hepatol* 2002, **17**:1331-1333.

5. Gaastra W, Kusters JG, van Duijkeren E, Lipman LJ: ***Escherichia fergusonii***. *Vet Microbiol* 2014, **172**(1-2):7-12.

6. Drzewiecka D: **Significance and Roles of *Proteus* spp. Bacteria in Natural Environments**. *Microb Ecol* 2016, **72**(4):741-758.

7. Dantas Lopes LD, Davis EW, Pereira e Silva MC, Weisberg AJ, Bresciani L, Chang JH, Loper JE, Andreote FD: **Tropical soils are a reservoir for fluorescent *Pseudomonas* spp. biodiversity**. *Environ Microbiol* 2018, **20**:62-74.

8. Mauchline TH, Malone JG: **Life in earth -- the root microbiome to the rescue?** *Curr Opin Microbiol* 2017, **37**:23-28.

9. Andrews M, Andrews ME: **Specificity in legume-*Rhizobia* symbioses**. *Int J Mol Sci* 2017, **18**:705.

10. Byrd AL, Belkaid Y, Segre JA: **The human skin microbiome**. *Nat Rev Microbiol* 2018, **16**(3):143-155.

11. Wieser M, Denner EB, Kampfer P, Schumann P, Tindall B, Steiner U, Vybiral D, Lubitz W, Maszenan AM, Patel BK *et al*: **Emended descriptions of the genus *Micrococcus, Micrococcus luteus* (Cohn 1872) and *Micrococcus lylae* (Kloos *et al.* 1974)**. *Int J Syst Evol Microbiol* 2002, **52**(Pt 2):629-637.

12. Kloos WE, Schliefer KH: **Isolation and characterization of *Staphylococci* from human skin II. Descriptions of four new species: *Staphylococcus warneri, Staphylococcus capitis, Staphylococcus hominis,* and *Staphylococcus simulans***. *Int J Syst Bacteriol* 1975, **25**:62-79.

13. Samuel BS, Rowedder H, Braendle C, Felix MA, Ruvkun G: ***Caenorhabditis elegans* responses to bacteria from its natural habitats**. *Proc Natl Acad Sci USA* 2016, **113**(27):E3941-E3949.
