# Supplementary material for: Skin-penetrating nematodes exhibit life-stage-specific interactions with host-associated and environmental bacteria
Source: BMC Biol. 2021 Oct 7;19:221. doi: 10.1186/s12915-021-01153-7 (PMC8499433; doi:10.1186/s12915-021-01153-7)

Additional file 8: Fig. S7

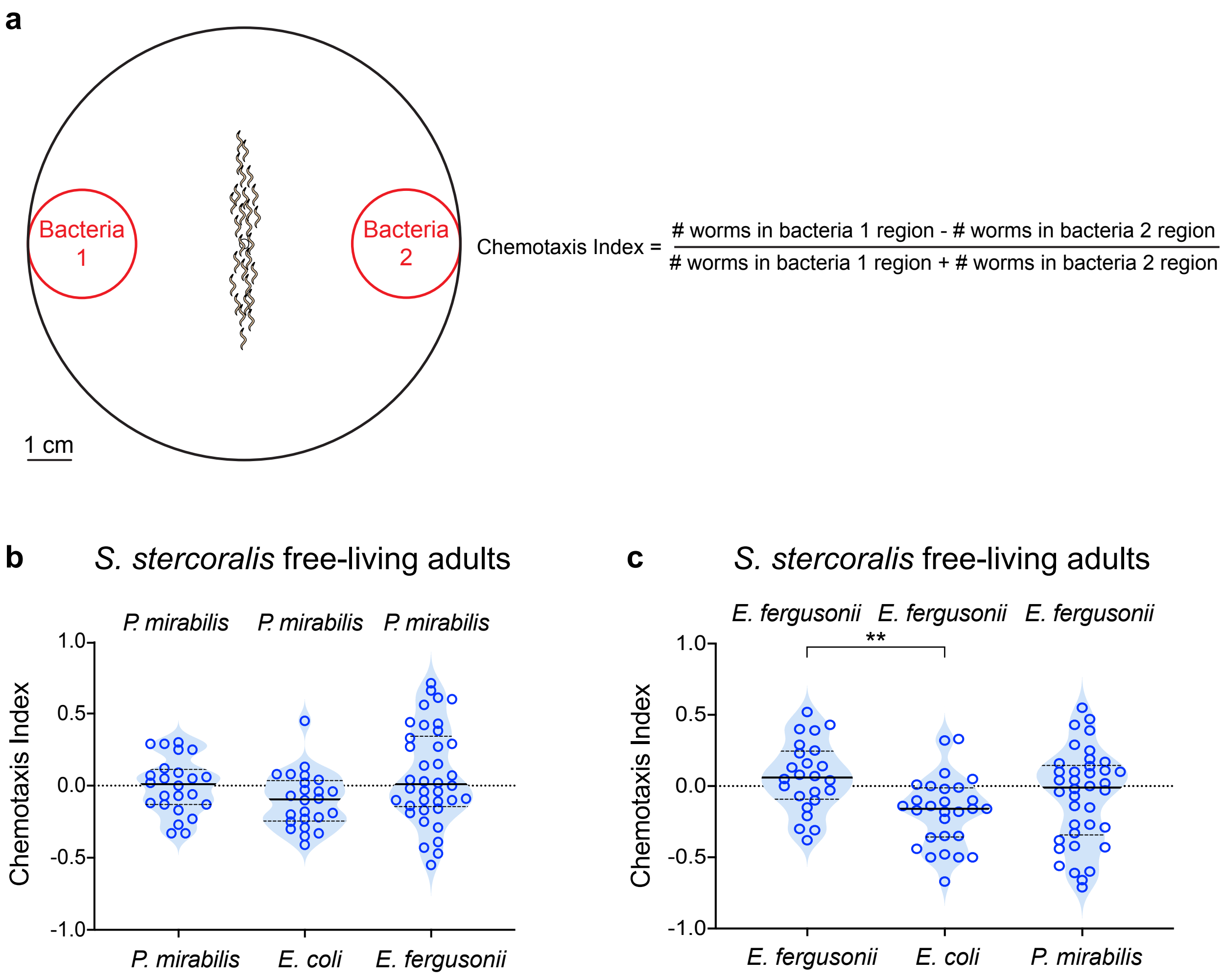

Supplement: Supplementary file 8 — Additional file 8: Fig. S7. S. stercoralis free-living adults do not display strong preferences among fecal/gut bacteria. a. Diagram of the bacterial chemotaxis assay. For bacterial competition chemotaxis assays, different bacteria were plated on each side of the plate. A chemotaxis index (CI) was calculated after counting the number of nematodes in each region as: CI = (# worms in bacteria 1 region - # worms in bacteria 2 region) / (# worms in bacteria 1 region + # worms in bacteria 2 region). b. S. stercoralis free-living adults did not prefer E. coli or E. fergusonii over P. mirabilis. No significant differences were detected (Brown-Forsythe and Welch ANOVA). c. S. stercoralis free-living adults showed a slight preference for E. coli over E. fergusonii. **p< 0.01, Brown-Forsythe and Welch ANOVA with Dunnett’s T3 post-test. Only the significant difference is noted. For b-c, each condition was compared to the control condition where the same bacterial species was plated on both sides of the plate. The same data are represented in b and c. n = 24-38 trials per condition, with 75-150 worms per trial. Each point in the graphs shows the chemotaxis index of a single trial; medians (solid lines) and interquartile ranges (dashed lines) are also shown. For each condition, the bacteria listed at the top and bottom of the graph indicate the two bacteria being tested in the competition assay. [file 12915_2021_1153_MOESM8_ESM.pdf]
